# Large-scale robot-assisted genome shuffling yields industrial *Saccharomyces cerevisiae* yeasts with increased ethanol tolerance

**DOI:** 10.1186/s13068-015-0216-0

**Published:** 2015-02-26

**Authors:** Tim Snoek, Martina Picca Nicolino, Stefanie Van den Bremt, Stijn Mertens, Veerle Saels, Alex Verplaetse, Jan Steensels, Kevin J Verstrepen

**Affiliations:** Laboratory for Genetics and Genomics, Centre of Microbial and Plant Genetics (CMPG), KU Leuven, Kasteelpark Arenberg 22, 3001 Leuven, Belgium; Laboratory for Systems Biology, VIB, Bio-Incubator, Gaston Geenslaan 1, 3001 Leuven, Belgium; Laboratory of Enzyme, Fermentation and Brewing Technology, KU Leuven technologiecampus Ghent, Gebroeders De Smetstraat 1, 9000 Ghent, Belgium

**Keywords:** Genome shuffling, Bioethanol, Ethanol tolerance, Breeding, Heterosis, Hybridization, Complex phenotype, Evolutionary engineering, Very high gravity, Fermentation

## Abstract

**Background:**

During the final phases of bioethanol fermentation, yeast cells face high ethanol concentrations. This stress results in slower or arrested fermentations and limits ethanol production. Novel *Saccharomyces cerevisiae* strains with superior ethanol tolerance may therefore allow increased yield and efficiency. Genome shuffling has emerged as a powerful approach to rapidly enhance complex traits including ethanol tolerance, yet previous efforts have mostly relied on a mutagenized pool of a single strain, which can potentially limit the effectiveness. Here, we explore novel robot-assisted strategies that allow to shuffle the genomes of multiple parental yeasts on an unprecedented scale.

**Results:**

Screening of 318 different yeasts for ethanol accumulation, sporulation efficiency, and genetic relatedness yielded eight heterothallic strains that served as parents for genome shuffling. In a first approach, the parental strains were subjected to multiple consecutive rounds of random genome shuffling with different selection methods, yielding several hybrids that showed increased ethanol tolerance. Interestingly, on average, hybrids from the first generation (F1) showed higher ethanol production than hybrids from the third generation (F3). In a second approach, we applied several successive rounds of robot-assisted targeted genome shuffling, yielding more than 3,000 targeted crosses. Hybrids selected for ethanol tolerance showed increased ethanol tolerance and production as compared to unselected hybrids, and F1 hybrids were on average superior to F3 hybrids. In total, 135 individual F1 and F3 hybrids were tested in small-scale very high gravity fermentations. Eight hybrids demonstrated superior fermentation performance over the commercial biofuel strain Ethanol Red, showing a 2 to 7% increase in maximal ethanol accumulation. In an 8-l pilot-scale test, the best-performing hybrid fermented medium containing 32% (*w*/*v*) glucose to dryness, yielding 18.7% (*v*/*v*) ethanol with a productivity of 0.90 g ethanol/l/h and a yield of 0.45 g ethanol/g glucose.

**Conclusions:**

We report the use of several different large-scale genome shuffling strategies to obtain novel hybrids with increased ethanol tolerance and fermentation capacity. Several of the novel hybrids show best-parent heterosis and outperform the commonly used bioethanol strain Ethanol Red, making them interesting candidate strains for industrial production.

**Electronic supplementary material:**

The online version of this article (doi:10.1186/s13068-015-0216-0) contains supplementary material, which is available to authorized users.

## Background

With an annual production of over 50 billion liters per year, bioethanol has become a valuable alternative for non-renewable fossil fuels, yet increases in productivity can aid in further establishing this biofuel. The yeast *Saccharomyces cerevisiae* is often the preferred micro-organism in ethanolic fermentations, due to its high natural ethanol tolerance and excellent fermentation capacity [1]. Very high gravity (VHG) fermentations, in which high (>270 g/l) initial sugar concentrations are used, can in theory yield much improved efficiency because higher final ethanol concentrations are reached [2]. However, stress factors encountered during the fermentation, especially ethanol stress, negatively impact the viability and fermentative performance of the yeast cells, which leads to sluggish or stuck fermentations [3]. Therefore, novel strains with improved ethanol tolerance may further increase productivity by allowing higher initial sugar concentrations that yield increased final ethanol concentrations.

Improving the ethanol tolerance of yeast is not straightforward. First, ethanol tolerance is a complex phenotype, which is underscored by transcriptome and single-gene knockout studies of ethanol-exposed strains that identified hundreds of genes with very different functions to be associated with ethanol tolerance (reviewed by Ma and Liu [4]). Although these studies have shed some light on the mechanisms of ethanol tolerance, detailed mechanistic insight explaining how and why cells can resist high ethanol concentrations is still lacking. Moreover, it is not clear if and how results obtained in laboratory strains, which are in general more ethanol sensitive, can be extrapolated to inherently more robust industrial strains [5]. In addition, there is no strict definition for ethanol tolerance and it is unclear how the different methods used to measure ethanol tolerance relate to the stress encountered in industrial fermentations [6].

Because of the complexity of ethanol tolerance, rational methods for strain improvement, such as genetic engineering, have only yielded limited success to improve the ethanol tolerance of industrial yeasts. By contrast, approaches that generate artificial variation in a non-targeted fashion, for instance by means of evolutionary engineering or mutagenesis, have proven more successful to improve this complex phenotype [7]. In the last decade, genome shuffling was added to the arsenal of techniques to enhance complex phenotypes in microbes without the need for insight into the molecular mechanisms governing the trait of interest (reviewed by Biot-Pelletier and Martin [8]). Genome shuffling consists of three steps [9]. First, a genetically diverse population is generated; mostly by induction of mutations in a single strain, for instance by using chemicals or UV radiation. Second, this diverse population is subjected to screening or selection in order to identify or enrich for the best-performing mutants. Third, the genomes of these superior cells are shuffled by means of asexual (protoplast fusion) or sexual (sporulation and mating) hybridization. In this way, novel combinations of beneficial mutations can be created and/or deleterious mutations can be removed. This whole procedure is typically repeated several times in order to further improve the phenotype, and thereby, genome shuffling allows for much larger leaps in the fitness landscape as compared to classical strain improvement by means of mutagenesis and screening [9]. Another advantage of genome shuffling is that this method is not considered to be genetic modification, in contrast to methods such as protoplast fusion. Therefore, novel strains generated by genome shuffling can be readily applied anywhere in the world without complex procedures to obtain approval to use the strains, even for the production of ethanol that would be used in foods and beverages. Multiple industrially relevant phenotypes in various micro-organisms, including tolerance to industrial stress factors as well as production titers, were successfully improved using genome shuffling [8].

Genome shuffling has also yielded yeast strains with improved ethanol tolerance and fermentation performance (see for an overview [7]). Whereas all these studies report an improvement of the parental strains, the industrial relevance of the novel strains is not always clear. Some researchers used laboratory strains [10,11], which likely do not perform well under industrial conditions. In other studies, the novel hybrids were not tested on a pilot scale [12-15]. Moreover, genome shuffling is often combined with genetic engineering [16,17], making some industrial applications problematic. In addition, most studies used only a limited number of different strains or relied on artificial diversity created by mutagenesis of a single strain. In this way, the vast potential of the rich natural genetic diversity of *Saccharomyces* yeasts is largely ignored. Moreover, mutagenesis may yield mutations that increase certain selected parameters, but negatively affect others. Even though such problems may also arise when combining naturally occurring mutations, in theory, these mutations have withstood the test of natural selection, increasing the chance that they do not negatively affect fitness. However, using naturally occurring mutations is more complex because it requires finding suitable parental strains that combine different positive mutations and also show sufficient sporulation efficiency and spore viability to allow genome shuffling, which is often a problem for industrial strains.

Here, we set out to characterize a large collection of *Saccharomyces* yeasts to identify eight parental strains that could be used for genome shuffling aimed at obtaining novel yeast hybrids that show increased ethanol tolerance compared to the currently used industrial yeasts, such as Ethanol Red. We explored different genome shuffling strategies and selection methods, including repeated rounds of random mating and robot-assisted targeted mating with selection for outcrossing. Our efforts resulted in several hybrids showing best-parent heterosis for ethanol production. One of those hybrids was tested in pilot-scale fermentations and retained its superior performance in these semi-industrial conditions, making it an interesting candidate for commercial bioethanol production.

## Results

### Large-scale screening of *Saccharomyces* yeasts for ethanol production and sporulation

Because we aimed to exploit the natural variation among *Saccharomyces* yeasts to create novel hybrids with increased ethanol tolerance, we screened a collection consisting of 318 *Saccharomyces* strains isolated from different natural niches and industries such as wine, ale, or biofuel production (see ‘Methods’). We first determined the ethanol accumulation capacity under lab-scale VHG conditions (fermentation of 150 ml YP + 35% (*w*/*v*) glucose). The average ethanol production varied widely among strains from different industries, with strains used for bioethanol production showing the highest average production, followed by wine yeasts, wild isolates, sake yeasts, spirits yeasts, and beer and bakery strains (see Additional file 1: Figure S1A). The strain showing the highest ethanol titer was Ethanol Red (Fermentis, S. I. Lesaffre) (Additional file 1: Figure S1B), a strain commonly used in first-generation bioethanol production.

Since many industrial *S. cerevisiae* strains are known to show poor sexual reproduction, we tested the capacity of all 318 yeasts to generate viable spores. We found that 172 (54%) of the strains formed spores, yet the sporulation efficiency and spore viability varied both within and among strains from different origins (Additional file 1: Figure S2A). Most of the lager, sake, and ale strains did not show sporulation, confirming previous studies reporting low sporulation efficiency among industrial strains [18,19]. Next, we determined the spore viability of each sporulating strain by dissecting four tetrads and observed that 144 strains yielded at least one viable spore (Additional file 1: Figure S2B). The majority (74/144) of the strains were found to be homothallic, whereas 46 strains gave rise to both haploid and diploid colonies after tetrad dissection. Twenty-three strains were found to be heterothallic and only produced colonies with a single mating-type. Only 12 of these heterothallic strains consistently showed 2a:2α segregation of the mating-type locus (Additional file 1: Figure S2C).

**Figure 1:**
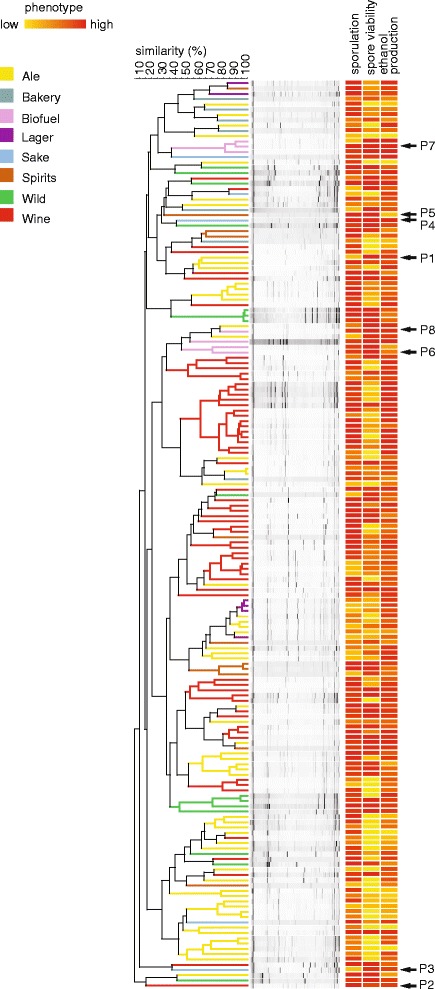
**Large-scale genotypic and phenotypic screening of** ***Saccharomyces*** **yeasts to select parental strains for genome shuffling.** Strains that show good sporulation were clustered according to their genetic relatedness as estimated by interdelta fingerprinting (see ‘Methods’). Phenotypes shown include sporulation capacity, spore viability, and ethanol production from a VHG substrate. Each of these phenotypes was measured for all the strains, and the measurements were then normalized and displayed using a color scale (heat map) as indicated in the figure. Eight genetically divergent heterothallic strains were selected that could sporulate and displayed high spore viability (indicated with arrows and codes P1-P8). This strain set included the commonly-used bioethanol strain Ethanol Red (P7); the best-fermenting strain out of the collection.

We hypothesized that shuffling the genomes of genetically divergent strains increases the chance of obtaining hybrids that show best-parent heterosis for our traits of interest (because each strain may contribute different beneficial genetic variation). To estimate the genetic relatedness of the different strains, we performed interdelta PCR fingerprinting on all 318 yeasts [20,21]. Figure 1 shows a heat map that represents the results of the phenotypic and genotypic screen.

Eight genetically divergent heterothallic strains showing high spore viability were selected as parental strains for genome shuffling. This set included Ethanol Red (P7), the best-performing strain from the screening for VHG fermentation capacity described above. Given its widespread commercial application in bioethanol fermentations, Ethanol Red is used as a reference strain throughout this paper. The other seven strains included a sake strain (P4) that showed a very high maximal ethanol accumulation, but fermented a bit slower than Ethanol Red, three strains (P1, P6, P8) that showed efficient fermentation during the first phase of the fermentation but reached lower final ethanol titers, two strains (P2, P3) that did not show exceptional fermentation efficiency, but showed efficient growth in the presence of ethanol (see later), and one strain (P5) that reached a lower final ethanol titer (Additional file 1: Table S1). This last strain was chosen because previous studies found that weak strains often do contain some beneficial genetic variation that is absent from superior strains [5,22,23].

### Growth in the presence of ethanol is correlated with fermentation performance

Genome shuffling typically produces a large pool of hybrids, many of which are inferior to the parental strains. Selecting the few superior hybrids from this pool is therefore not always straightforward, especially for traits like ethanol production, which cannot be tested in bulk. Therefore, we explored the correlation between ethanol production and the ability to grow in the presence of high concentrations of ethanol, a trait that is easier to select for. We measured the growth capacity of each strain on solid YPD supplemented with different ethanol concentrations (5 to 14% (*v*/*v*)) using a robot-assisted screen (see ‘Methods’). We found a significant positive correlation between production and growth in the presence of ethanol, with the strongest correlation in medium supplemented with 11% (*v*/*v*) ethanol (Pearson *r* = 0.51, *P* < 0.0001, Additional file 1: Figure S3). Hence, we decided to pre-select hybrids generated by genome shuffling for their capacity to grow in the presence of ethanol, and then test individual hybrids for their capacity to produce high concentrations of ethanol (see further). In addition, we used growth in the presence of ethanol as a selection method between different rounds of genome shuffling in some approaches.

### Genome shuffling based on random mating generates improved F3 hybrids

**Figure 2:**
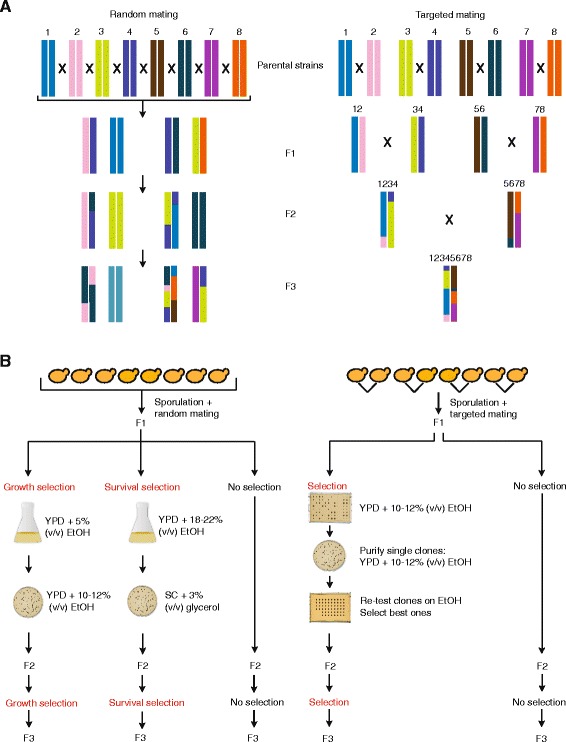
**Two different genome shuffling strategies used to generate hybrids starting from the eight parental strains. (A)** Conceptual outline of targeted and random genome shuffling. Genome shuffling based on random mating (left) allows random mating between spores form the eight parental strains (F1) and between spores derived from genome-shuffled hybrid populations (F2 and F3). Genome shuffling based on targeted mating (right) exploits the selection of true outcrossed hybrids using plasmid-based markers at each stage, ensuring the presence of the eight initial genomes in the final F3 hybrids. For simplicity, homologous chromosomes of the parental strains have been given the same color, although parental strains were heterozygous diploids. **(B)** Details of the experimental procedures used for genome shuffling strategies. Genome shuffling based on random mating (left) was performed using two different types of selection after each round of mating. First, hybrids were selected for their capacity to grow in the presence of ethanol by first inoculating them into medium containing 5% (*v*/*v*) ethanol followed by growth in the presence of 10 to 12% (*v*/*v*) ethanol (referred to as ‘growth selection,’ similar to the selection applied for targeted mating). Alternatively, the hybrids were selected for survival in medium containing very high (18 to 22% (*v*/*v*)) ethanol levels (‘survival selection’). In parallel to these two approaches, we also carried out shuffling without any selection in between the different rounds of hybridization (‘no selection’). For targeted mating (right), a robot is used to perform specific crosses between the eight parental strains in all pairwise combinations, followed by screening for ethanol tolerance (growth capacity in the presence of ethanol). The best-performing hybrids were used as parental strains for the next round of robot-based targeted mating, and in parallel similar breeding schemes were carried out without applying any selection after each round of shuffling. See ‘Methods’ and Additional file 1: Figure S5 for more details about these procedures.

Starting from the eight selected parental strains, we created several pools of genome-shuffled hybrids using two different strategies. First, we used random genome shuffling, where the eight parental strains were allowed to interbreed randomly. In a second approach, we used large-scale robot-assisted targeted genome shuffling, where specific pairs of strains were mated (see ‘Genome shuffling based on targeted mating yields hybrids with increased ethanol tolerance’ and Figure 2A). For each of these strategies, we performed three rounds of shuffling, thereby generating three generations of genome-shuffled pools of hybrids (referred to as F1, F2, and F3).

For the random genome shuffling strategy, spores derived from the eight parental strains were subjected to mass mating (see ‘Methods’) to create a heterogeneous F1 mixture. We used three different selection methods to obtain F3 hybrids starting from this F1 generation. First, we selected the pool of hybrids for growth in the presence of ethanol after each round of shuffling, thereby exploiting the correlation between ethanol production and growth in high ethanol conditions (see above). In a second approach, the hybrids generated after each round of shuffling were selected for survival in medium containing extremely high ethanol levels. Third, we also performed the different rounds of random genome shuffling in the absence of any selection. Figure 2B shows a schematic overview of these different genome shuffling strategies and selection methods.

**Figure 3:**
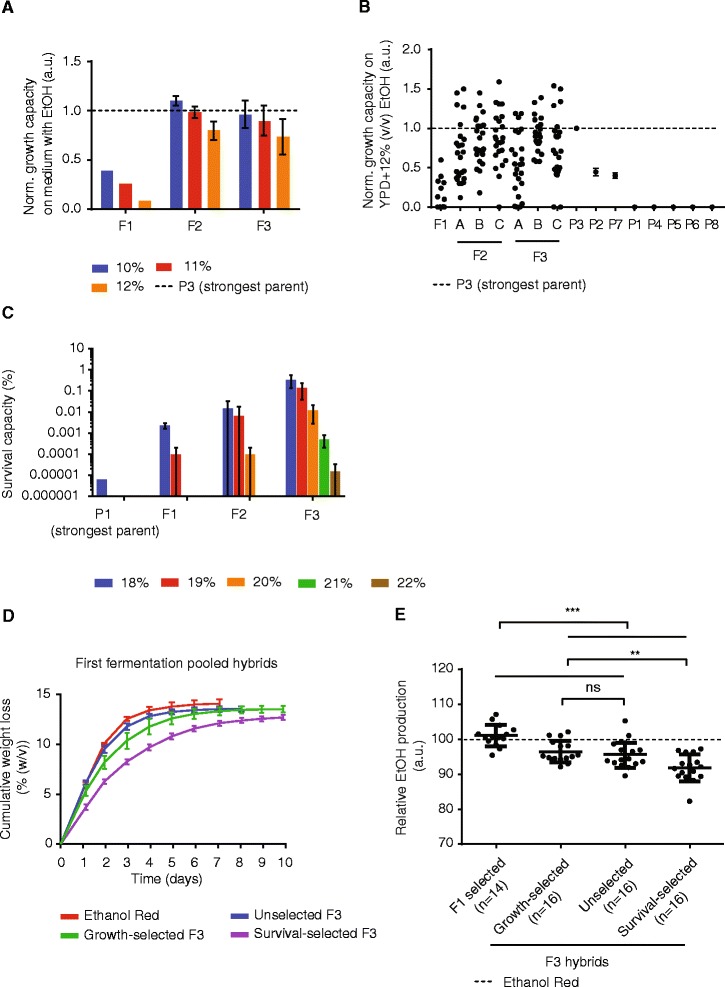
**Genome shuffling based on random mating generates hybrids with increased ethanol tolerance. (A)** Growth capacity in the presence of ethanol of clones obtained before selection (F1), after one round (F2) and two rounds of shuffling and selection (F3). Whereas F2 hybrids show a clear increase in growth capacity, F3 hybrids do not show further increases. **(B)** Same data as Figure 3A for the individual isolates at 12% (*v*/*v*) ethanol and the parental strains (*n* = 2 for each parent). F2 and F3 isolates on average performed better than F1 isolates, but did not show statistical differences between different replicates and generations (unpaired *t*-test *P* > 0.05). A, B, and C are biological replicates. **(C)** Selection for survival in high ethanol yields hybrids with increased survival capacity which further increases after each round of shuffling. Each bar represents the average and standard deviation of three biological replicates, except for F1 19% (*v*/*v*) where *n* = 2. Survival after exposure to 21% (*v*/*v*) and 22% (*v*/*v*) was only measured for F3 populations. The data for the strongest parental strain (P1) is displayed for 18% (*v*/*v*); this strain completely lost its viability after exposure to 19% (*v*/*v*). **(D)** Fermentation performance of F3 populations in YP + 35% (*w*/*v*) glucose. The cumulative weight loss, a proxy for CO_2_ production, during the fermentations is shown. Each line represents the average of two replicate fermentations for the reference strain (Ethanol Red), six replicates for growth-selected and survival-selected populations, or twelve replicates for unselected populations. Error bars represent standard deviations. **(E)** Selected isolates from several pools of hybrids were tested for their maximal ethanol accumulation in YP + 35% (*w*/*v*) glucose. On average, F1 hybrids showed the highest average ethanol production levels. Unselected and growth-selected F3 hybrids on average performed similar, but better than survival-selected F3 hybrids. The dotted line indicates the ethanol production of Ethanol Red. Unpaired *t*-test: ns, not significant; **P ≤ 0.01; ***P ≤ 0.001.

Random genome shuffling using selection for growth in the presence of ethanol was carried out for three consecutive rounds of shuffling in three biological replicates (see ‘Methods’ and Additional file 1: Table S2). To directly compare the ethanol tolerance of populations obtained after different rounds of shuffling, we tested from each biological replicate of the F2 and F3 generations as well as from the unselected F1 pool 24 random isolates for their capacity to grow on medium containing 10 to 12% (*v*/*v*) ethanol (Figure 3A and Additional file 1: Table S2). We found that the ethanol tolerance increased after one round of shuffling and selection, but that another round did not increase the tolerance further (Figure 3A). Multiple clones (29/72 from F2, 21/72 from F3) showed best-parent heterosis (that is, they showed higher ethanol tolerance than all parental strains) (Figure 3B).

The second selection method during random genome shuffling was based on survival in high ethanol levels (18 to 22% (*v*/*v*)). Although the ethanol tolerance of microbes is often measured by the capability to survive exposure to lethal ethanol levels, this is the first study using this phenotype as a selection method in genome shuffling experiments. After each round of shuffling, pools of hybrids were grown to the late log-phase, and from each pool, 10^9^ cells were harvested and exposed to rich medium supplemented with ethanol. After incubation for 16 h, cells were plated on solid glycerol medium to select for cells that survived exposure to ethanol and that were still able to respire (since ethanol is a potent inducer of so-called petite mutations that lead to a loss of mitochondrial function [24]). We carried out this selection method in three biological replicates. After the first round of shuffling, we selected F1 hybrids that survived 18% (*v*/*v*) ethanol, and after the second round of shuffling, F2 hybrids were selected that survived exposure to 19% (*v*/*v*) ethanol (see ‘Methods’ and Additional file 1: Table S3). This selection strategy was highly selective: whereas 10^9^ cells were exposed to ethanol, only about 30,000 cells (approximately 0.003% of the population) were recovered for the F1 generation after exposure to 18% (*v*/*v*) ethanol (Additional file 1: Table S3). For each generation, we also tested the capacity of the hybrids to survive a range of ethanol concentrations and found that the survival capability increased with each round of shuffling, indicating that we were successfully selecting cells with improved tolerance for high ethanol concentrations (Figure 3C and Additional file 1: Table S3). Moreover, subjecting unselected and growth-selected F3 populations to high ethanol levels gave results similar to or worse than those obtained for F1 populations, indicating that the increased survival capacity resulted from the selection and not merely from repeated rounds of sporulation and mating (see Additional file 1: Table S4).

### Different selection methods yield hybrids with different fermentation performance

To investigate how the different selection methods used in our random genome shuffling experiments (selection for growth, survival, or no selection) affected the performance of the final F3 populations in VHG fermentations, we inoculated subpopulations (that is, samples of the pools of hybrids and not pre-selected pure individual hybrids) of the different F3 populations into YP medium supplemented with 35% (*w*/*v*) glucose. None of the hybrid populations fermented faster or more completely than the control strain Ethanol Red. Interestingly, among hybrid populations, unselected populations showed the fastest fermentation, whereas growth-selected populations fermented to a same degree, but more slowly (Figure 3D). On the other hand, survival-selected populations showed less efficient fermentation. Re-inoculating the cells for two subsequent fermentations confirmed these differences (Additional file 1: Figure S4). After three subsequent fermentations, we selected 16 colonies from populations derived from each genome shuffling strategy. In addition, we also selected 14 different F1 clones that showed a high growth capacity in the presence of ethanol. We evaluated the fermentation performance of all these isolates by measuring the maximal ethanol accumulation in VHG fermentations, and normalized this value to the average ethanol level produced by Ethanol Red in the same batch. Interestingly, on average, individual hybrids selected from the F1 population showed superior fermentation performance compared to the hybrids isolated from the F3 pools, with 10 out of 14 F1 hybrids reaching a higher final ethanol level than Ethanol Red (Figure 3E). There was no statistical difference in the final ethanol titer between growth-selected and unselected F3 hybrids, whereas all categories performed statistically better than survival-selected hybrids (Figure 3E and Additional file 2). In this screening, three growth-selected F3 hybrids and two unselected F3 hybrids showed a higher maximal ethanol accumulation than Ethanol Red (relative ethanol production >100).

### Genome shuffling based on targeted mating yields hybrids with increased ethanol tolerance

**Figure 4:**
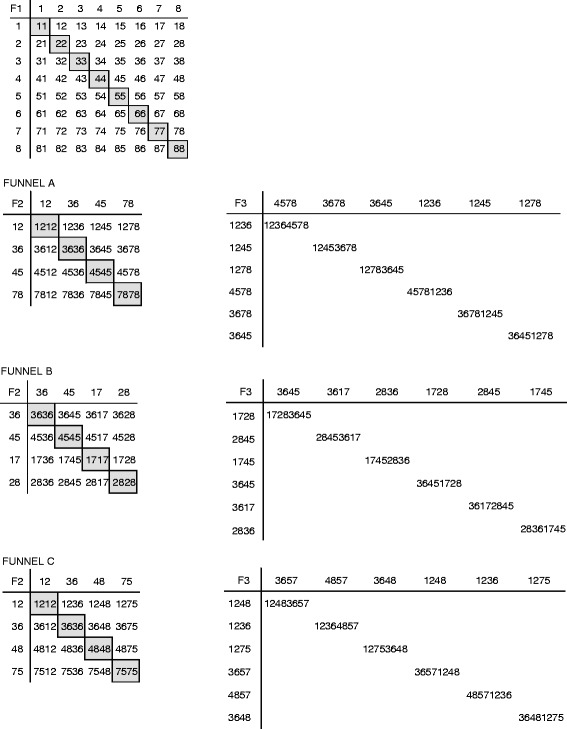
**Schematic overview of targeted genome shuffling.** For the first round of targeted genome shuffling (creating pools of F1 hybrids), the eight parental strains (numbered 1 to 8) were crossed in all pairwise combinations, including inbreeding. After screening and selection (see main text), isolated F1 hybrids were used to create three funnels (A, B, C). In each funnel, the F1 hybrids were crossed in all pairwise combinations to make F2 hybrids, followed by screening and selection for ethanol tolerance. For the last round of genome shuffling, F2 hybrids were crossed in such a way that F3 hybrids incorporated genetic material from all eight initial parental strains. Note that these funnel schemes were also carried out for F1 hybrids from the same parental strains that were not selected for growth in the presence of ethanol, and for which no selection was performed in between the different rounds of hybridization.

In addition to the random genome shuffling strategy described above, we mated the same eight parental strains using a targeted funnel-breeding scheme based on non-stable plasmid-based dominant markers (see Figure 2A and ‘Methods’). This strategy involved three rounds of shuffling; F1 hybrids incorporated two of the initial genomes; F2 hybrids incorporated four parental genomes; and finally, F3 hybrids were a mosaic of the eight parental genomes (Figures 2A and 4). We carried out two parallel experiments: one in which we performed selection for growth in the presence of ethanol after each round of shuffling and one experiment that started from unselected F1 hybrids and in which no intermediate selection was carried out (Figure 2B).

**Figure 5:**
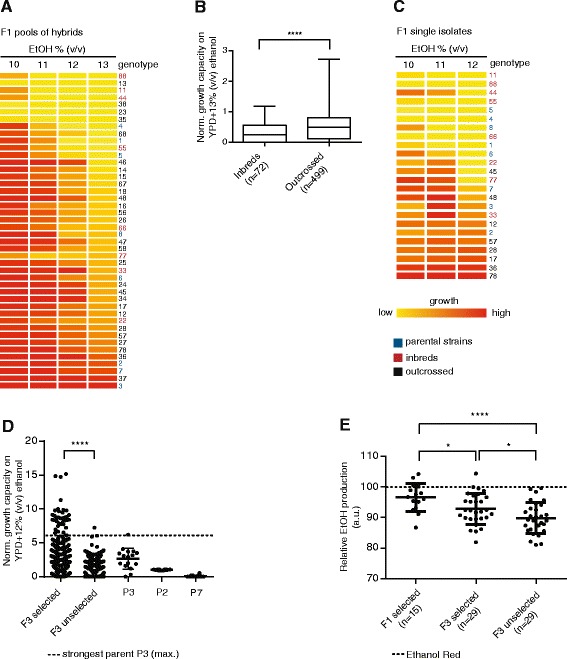
**Genome shuffling based on targeted mating yields hybrids with increased growth and fermentation capacity. (A)** The eight parental strains were crossed in all pairwise combinations. Each horizontal line represents the average growth in medium with different concentrations of ethanol for all hybrid populations, as well as the parental strains. The data was normalized and converted to a heat map, and strains were ranked from low to high based on their growth in 13% (*v*/*v*) ethanol. **(B)** Outcrossed F1 hybrids display higher ethanol tolerance than F1 inbreds (Mann-Whitney test: ****P ≤ 0.0001). **(C)** Most isolated F1 outcrossed hybrids show heterosis for ethanol tolerance, whereas most inbreds perform poorer than their parents (‘inbreeding depression’). Each horizontal line represents the average performance of all single clones from a certain cross, inbred or parental strain on different concentrations of ethanol. Strains were ranked from low to high based on 12% (*v*/*v*) ethanol data. **(D)** F3 hybrids subjected to selection for ethanol tolerance (measured by the capacity to grow in the presence of ethanol) after each round of genome shuffling show higher ethanol tolerance than unselected F3 hybrids (Mann-Whitney test: ****P ≤ 0.0001). The dot plots show the relative growth of all F3 isolates, obtained with and without selection after each round of shuffling, respectively, on 12% (*v*/*v*) ethanol. The data from the strongest parental strains (P3, P2, and P7) are shown for comparison. **(E)** Individual hybrids show different maximal ethanol accumulation in VHG fermentations. F1 hybrids selected for their capacity to grow in the presence of high ethanol levels on average show the highest ethanol production. Next, F3 hybrids subjected to selection after each round of shuffling on average show higher ethanol production that unselected F3 hybrids. The dotted line indicates the ethanol production level of Ethanol Red. Unpaired *t*-test: *P ≤ 0.05; ****P ≤ 0.0001.

To generate a large number of F1 hybrids with known ancestry, we crossed the eight parental strains in all pairwise combinations in multiple replicates by placing spore suspensions of strains carrying different antibiotic resistance markers in close proximity on solid rich medium using a robot (see Additional file 1: Figure S5 and ‘Methods’ for details). We obtained 500 outcrossed and 72 inbred F1 pools of hybrids, covering all possible crossings at least once. We assayed the growth of these pools of outcrossed and inbred F1 hybrids as well as the parental strains in the presence of different concentrations of ethanol as well as on medium without ethanol using a robot-based spotting assay (Additional file 1: Figure S6). Next, the ethanol tolerance values of all pools derived from a certain cross were averaged per tested ethanol concentration, and ranked based on the highest ethanol concentration measured (13% (*v*/*v*); see Figure 5A). A striking result was that the average ethanol tolerance of outcrossed hybrids was significantly higher than that of inbreds (Figure 5B and Additional file 1: Figure S7).

As a next step, we purified and re-tested single isolates from eight different F1 populations that showed high ethanol tolerance as well as from inbred populations from each parental strain. We found that isolated inbreds performed worse than their respective parents except for strain P3, whereas seven outcrossed F1 strains showed best-parent heterosis and one F1 hybrid performed in between its respective parents, underscoring the large phenotypic improvement reached by outcrossing (Figure 5C). Eight highly ethanol-tolerant F1 clones were used to create three funnel breeding schemes for the next round of genome shuffling based on targeted mating (see Figure 4 for details). In total, we assayed the ethanol tolerance of 720 outcrossed and 236 inbred pools of hybrids for the F2 generation, and we found that, similar to the first round of targeted mating, the ethanol tolerance of outcrossed F2 hybrids was higher than the ethanol tolerance of inbreds (Additional file 1: Figure S8). Per funnel, we isolated six F2 isolates that showed high ethanol tolerance. For the final round of mating, we crossed the selected F2 hybrids of each funnel in such a way that F3 hybrids incorporated the eight parental genomes. In parallel, these exact procedures, that is, the same targeted mating schemes consisting of multiple rounds of shuffling, were carried out using unselected F1 hybrids without intermediate selection for ethanol tolerance.

In order to compare the ethanol tolerance of selected and unselected F3 hybrids, we assayed the ethanol tolerance for 784 selected pools of F3 crosses and 596 pools of unselected F3 crosses. Next, we isolated 310 clones (10 single colonies from 31 pools) for the funnels where selection was applied after each round of mating and another 310 clones for funnels where no selection was applied in between each round mating, and tested the growth capacity of these pure isolates in the presence of ethanol. As expected, we found that selected hybrid isolates grew better than unselected hybrids (Figure 5D) on ethanol, and some showed best-parent heterosis and performed better than the strongest parental strain.

### On average, targeted F1 hybrids show superior VHG fermentation over targeted F3 hybrids

To find out whether increased ethanol tolerance leads to increased maximal ethanol accumulation in VHG fermentations, we tested the best-performing clones of selected and unselected targeted F3 hybrids in lab-scale VHG fermentations. In addition, we included 15 selected targeted F1 hybrids (the eight F1 isolates used in the funnels plus seven additional isolates). Interestingly, F1 hybrids on average showed higher ethanol production than both selected and unselected F3 hybrids (Figure 5E). This may be partly explained by the presence of a larger fraction of the Ethanol Red genome in these F1 strains (Additional file 1: Figure S9). In addition, selection for ethanol tolerance after each round of shuffling yielded F3 hybrids that show a higher ethanol production than those obtained from crosses where no selection was applied until after the last round of genome shuffling, indicating that selection for ethanol tolerance can in some cases increase the chances of selecting hybrids with increased ethanol production. Overall, in this screening, we identified four F1 hybrids and one F3 hybrid that showed a higher ethanol production than Ethanol Red (Additional file 3).

### Confirmation of the increased fermentation capacity of random and targeted hybrids

To confirm that some hybrids obtained in our different genome shuffling experiments outperform the commonly used bioethanol strain Ethanol Red, we re-tested thirteen hybrids (four F1 and four F3 hybrids from the ‘random’ approach and four F1 and one F3 hybrid from the ‘targeted’ approach; see Additional files 2 and 3) in triplicate in small-scale VHG fermentations. In total, eight hybrids (H1-H8) showed a statistically significant higher maximal ethanol accumulation (unpaired *t-*test: *P* < 0.05) than Ethanol Red (Additional file 4). Since it took these hybrids longer to complete the fermentation than Ethanol Red, we calculated an estimated ethanol productivity after 7 days (when Ethanol Red finished the fermentation). Seven hybrids (H1-H7) showed a significantly higher ethanol production rate, whereas the eighth hybrid (H8) fermented more slowly (Table 1). The average ethanol production for four hybrids (H9-H12) was statistically identical to Ethanol Red, yet one of those hybrids (H9) completed the fermentation 1 day faster than Ethanol Red (see Additional file 4 and Table 1). One hybrid (H13) showed a lower maximal ethanol accumulation than Ethanol Red. For the improved hybrids, the ethanol yield (grams of ethanol produced per grams of sugar consumed) was not statistically different from the yield of Ethanol Red (0.49 g/g), indicating that these hybrids ferment with an efficiency similar to Ethanol Red (Additional file 4).

**Table 1:** **Hybrids with improved fermentation performance identified in this study**

			**Ethanol production**		**Estimated ethanol productivity** ***d*** **= 7**			**Yield**
**Code**	**Genotype**	**Generation**	**Relative**	**(% (** ***v*** **/** ***v*** **))**	**(g/l)**	**Relative**	**(g/l/h)**	**(g/g)**
H1	47	F1	107.3 ± 1.16	20.0 ± 0.22	158.1 ± 1.70	105.7 ± 1.27	0.94 ± 0.01	0.49 ± 0.00
H2	Unknown	F1	107.3 ± 0.21	20.0 ± 0.04	158.2 ± 0.32	106.7 ± 0.19	0.95 ± 0.00	0.49 ± 0.00
H3	Unknown	F3	106.2 ± 1.16	19.8 ± 0.22	156.6 ± 1.71	103.6 ± 1.32	0.93 ± 0.01	0.49 ± 0.00
H4	Unknown	F1	105.4 ± 2.35	19.4 ± 0.44	153.2 ± 0.66	104.8 ± 1.89	0.94 ± 0.02	0.49 ± 0.00
H5	12364578	F3	105.0 ± 0.41	19.6 ± 0.08	154.8 ± 0.60	104.5 ± 0.31	0.93 ± 0.00	0.48 ± 0.00
H6	Unknown	F1	104.9 ± 1.01	19.6 ± 0.19	154.6 ± 1.49	104.7 ± 0.51	0.94 ± 0.00	0.49 ± 0.01
H7	Unknown	F1	103.9 ± 0.45	19.4 ± 0.08	153.2 ± 0.66	102.3 ± 0.45	0.91 ± 0.00	0.50 ± 0.01
H8	Unknown	F3	102.0 ± 0.70	19.0 ± 0.13	150.3 ± 1.03	98.3 ± 0.59	0.88 ± 0.01	0.49 ± 0.00
H9	78	F1	100.0 ± 1.05	19.1 ± 0.20	151.0 ± 1.58	NA	NA	0.48 ± 0.01
P7	EtOH Red	**-**	100 ± 0.83	19.0 ± 0.40	149.7 ± 2.94	100 ± 0.84	0.90 ± 0.03	0.49 ± 0.01

Genotype indicates the predicted genotype; for hybrids generated by random mating the genotypes are unknown, whereas for targeted hybrids the parental strains are indicated. Hybrids H1 up to H7 show both increased maximal ethanol accumulation and estimated ethanol productivity relative to Ethanol Red. Data for Ethanol Red is based on 30 biological replicates. See Additional file 4 for the complete dataset.

### Pilot-scale testing of one superior hybrid

**Figure 6:**
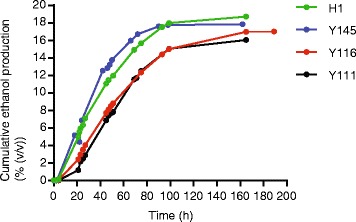
**A targeted hybrid (H1) generated by genome shuffling excels in pilot scale fermentations.** H1 and three high-ethanol producing industrial strains (Y145, Y116 and Y111) were each inoculated into 8 l VHG medium with 32% (*w*/*v*) glucose in a bioreactor. On regular time points the ethanol concentration was determined.

We set out to examine whether the performance of one of the best hybrids, H1, could be confirmed in semi-industrial conditions. First, we screened the fermentation capacity of H1 and 26 natural strains from the collection in static lab-scale fermentations (100 ml fermentation medium containing 320 g/l glucose) and measured the final ethanol production. Hybrid H1 ranked second in this screening, yet among the top strains, no statistical differences in ethanol titer could be detected (Additional file 5). Next, we tested the performance of H1 and the three other best-performing strains in 8 l VHG medium in a bioreactor. Notably, Ethanol Red was not tested on this scale since it ranked only ninth in the screening. H1 and Y145 showed superior fermentation performance over the other two strains (Figure 6); both strains completely utilized all the initial glucose, yet H1 showed the highest final ethanol titer, yield, and ethanol productivity (Table 2).

**Table 2:** **The performance of hybrid H1 and three high ethanol-producing strains on a pilot scale**

**Strain**	**Ethanol production (% (** ***v*** **/** ***v*** **))**	**Yield (%)**	**Yield (g/g)**	**Ethanol productivity (g/l/h)**
H1	18.7	88.8	0.45	0.90
Y145	17.9	86.5	0.44	0.87
Y116	17.0	88.8	0.45	0.71
Y111	16.0	88.0	0.45	0.77

Each strain was inoculated into 8 l of VHG medium. After 7 days, the fermentations were terminated, and the ethanol and glucose content were determined.

## Discussion

In this study we developed multiple large-scale genome shuffling strategies, which yielded several new hybrid yeasts that show improved maximal ethanol accumulation and ethanol tolerance compared to the commonly used biofuel strain Ethanol Red. Yeast strains with increased fermentation performance can help to increase the productivity and economic viability of biofuel production. A potential problem of genome shuffling is that many industrial strains show poor sexual reproduction and therefore are not suitable for strain improvement by means of mating. However, after screening more than 300 strains for their ability to form spores, we found the total number of strains that could potentially be used for genome shuffling to be sufficient to cover a large proportion of the existing natural genetic variation in *S. cerevisiae* (Figure 1).

The use of genome shuffling has already been applied to multiple phenotypes in *S. cerevisiae*, including tolerance to various stressors such as acetic acid [25], spent sulfite liquor [26], heat [27,28], ethanol, and fermentation capacity (as discussed by Steensels et al. [7]). In contrast to most previous genome shuffling experiments, we exploited the large natural variation among *Saccharomyces* yeasts. Instead of starting from one mutagenized strain, we selected eight parental strains, all shown to be genetically divergent heterozygous diploids (ongoing work in our laboratory by Brigida Gallone et al.). In addition, we used robotized assays that allowed us to generate and test many more hybrids than previous studies, thereby maximizing the chances of generating and finding superior hybrids.

Since we generated a huge number of different hybrids, testing each hybrid individually in a small-scale fermentation was impossible. Therefore, we used a robot-assisted screen for growth in the presence of a range of different ethanol concentrations (5 to 14% (*v*/*v*)), a phenotype for which we found significant correlations with ethanol production. Our results demonstrate that selecting for growth capacity in the presence of ethanol offers an efficient way to perform a first screening to select variants that are more likely to show increased fermentation capacity. Previously, Zheng et al. [12] explored the correlation between different industrially relevant phenotypes and did not find a significant correlation between growth in the presence of ethanol and ethanol production. However, we tested a much higher number of different yeast strains (308 versus 15). In addition, we found the highest correlation with growth in the presence of 11% (*v*/*v*) ethanol, a concentration not tested by Zheng et al. [12], suggesting that higher ethanol stress is more efficient for pre-screening or selecting strains for VHG fermentation. Therefore, we used selection for growth in the presence of ethanol as one of the selection methods between each round of genome shuffling. It is important to note that growth and production are at best weakly correlated, as also reported by Pais and colleagues [29], suggesting that both phenotypes have a partly different genetic basis.

Apart from yielding superior novel hybrid yeasts, our experiments also generated insight into the dynamics and efficiency of different genome shuffling strategies to obtain yeasts with improved ethanol tolerance and maximal ethanol production. We tested two different genome shuffling strategies, one based on random mating and one based on targeted mating. Moreover, for these two strategies, we compared the effects of selecting a subpopulation of superior hybrids in terms of ethanol tolerance after each round of shuffling, versus a parallel experiment in which we did not apply any selection until after the last round of shuffling (see further).

The first selection method encompassed selection for growth in the presence of ethanol after each round of shuffling. Interestingly, we found that, on average, F1 hybrids selected for their growth capacity in the presence of ethanol showed superior fermentation performance compared to F3 hybrids, both for genome shuffling with random mating and targeted mating. This can partly be explained by the finding that out of the eight selected parental strains, some specific combinations of parents provide a superior genetic basis, so that further crossing with other strains often results in weaker hybrids (see further). Another factor that might contribute is sign epistasis, which occurs when two alleles that each are beneficial in a particular genetic background result in a detrimental effect when combined in the same strain. Since F3 hybrids are expected to possess more complex mosaic genetic backgrounds, where alleles from different strains are combined, the effect of sign epistasis might be more pronounced. On the other hand, at least in theory, if sufficient F2 and F3 hybrids are tested, it should be possible to isolate hybrids that are superior to the best F1 hybrids, even if these superior F2 and F3 hybrids are very rare. Indeed, while superior F3 hybrids were more rare than improved F1 hybrids, we were able to isolate several superior F3 strains.

Since the targeted mating strategy allowed us to keep track of the ancestry of the hybrids and examine the contribution of the different parental genomes, we found that the targeted F1 hybrids that we tested in fermentations were considerably enriched in the Ethanol Red (P7) genome, and to a lesser degree in the P4 background, whereas the P1, P5, and P6 genomes seem under-represented (Additional file 1: Figure S9). The depletion of these genomes was perhaps not unexpected since we used growth in the presence of ethanol as a pre-selection method; P1, P5, and P6 grow relatively poorly in the presence of ethanol (Additional file 1: Figure S6). Indeed, all four targeted F1 hybrids that reached a higher final ethanol titer than Ethanol Red share Ethanol Red as one of their parents (Additional file 3).

Interestingly, we recovered only 1 (out of 29) targeted F3 hybrids selected for growth in the presence of ethanol that reached a higher ethanol titer than Ethanol Red. This seems to suggest that it may not be efficient to repeat the genome shuffling for several rounds and that it is instead better to stop after one round of shuffling. There are multiple possible explanations for the superior fermentation performance of (targeted) F1 compared to F3 hybrids. Firstly, some strains, such as Ethanol red and P4, already show excellent ethanol accumulation in VHG fermentation. Hence, it seems likely that these parents possess interesting combinations of alleles. It seems probable that at least some F1 hybrids inherit the majority of these beneficial alleles, but this chance decreases with each round of crossing. In theory, further rounds of crossing may result in the combination of beneficial alleles from more strains, but in reality, this would require generating and testing a very large number of hybrids. Second, the scatterplot showing the correlation between growth and ethanol production suggests that strains that grow weakly in the presence of ethanol are unlikely to produce high ethanol levels (Additional file 1: Figure S3B). Nevertheless, strains that grow well in the presence of ethanol are not always the best ethanol-producing strains, which was also illustrated by our parental strains: the best-fermenting strain out of the collection, Ethanol Red, grows well in the presence of ethanol, but not as good as P3, which produces considerably lower ethanol levels. The repeated selection for ethanol tolerance in consecutive rounds of genome shuffling may therefore exclude some of the high ethanol-producing strains.

In general, F1 hybrids generated by random mating also performed better than F3 hybrids. Specifically, 10 (out of 14) F1 hybrids from the random shuffling approach that were screened in VHG fermentations reached a higher ethanol titer than Ethanol Red, as compared to only 3 (out of 16) F3 hybrids. Based on these frequencies, genome shuffling using random mating followed by pre-selecting the F1 hybrids for their growth capacity in the presence of ethanol appears to be the best strategy to identify novel strains with increased fermentation performance. Nevertheless, we also confirmed the increased performance of a number of F3 hybrids from both strategies. For reasons described above, we hypothesize that although F3 hybrids with increased VHG performance were less frequent than superior F1 hybrids, these F3 pools might in theory contain hybrids that outperform some of the best F1 hybrids because the theoretical genetic diversity is larger in the F3 pool compared to the F1 pool.

For both genome shuffling based on random mating and on targeted mating, we also investigated the dynamics of not applying any selection in between the different rounds of genome shuffling. The idea behind not selecting the hybrids after each round of genome shuffling is that, in theory, it is possible that some F1 or F2 hybrids that show weak ethanol tolerance harbor beneficial (recessive) alleles that can aid in bringing forth superior hybrids. By selecting after each round of shuffling, such F1 and F2 hybrids would not be selected for the next round of crossing. However, our results demonstrate that on average, F3 hybrids that were obtained from genome shuffling schemes that involved selection for growth in the presence of ethanol after each round of crossing showed better ethanol tolerance and ethanol production than F3 hybrids that did not undergo selection after each round of shuffling.

In addition to selection for increased growth in the presence of high ethanol concentrations, we also explored a selection strategy based on survival in medium containing even higher ethanol levels. Ethanol tolerance has multiple definitions, including the ability to survive high ethanol concentrations [6,30], yet until this report, no study has used this phenotype as a selection method in genome shuffling experiments. The advantage of selecting for survival is that extremely large numbers of hybrids can be subjected to selection in bulk, which in theory should increase the chance of selecting the very best hybrids present in the population. This selection strategy proved to be quite powerful and efficient, leading to a stepwise improvement of the capacity to survive high ethanol levels with each round of shuffling and selection. In a control experiment, we found that genome-shuffled F3 populations that were subjected to selection for growth in the presence of ethanol after each round of shuffling as well as F3 populations resulting from genome shuffling schemes without selection did not show increased survival capacity when subjected to high ethanol levels (Additional file 1: Table S4). Actually, these populations performed similar to F1 populations, suggesting that growth capacity in the presence of ethanol and the capacity to survive high ethanol levels as measured in our experimental setups are not correlated. Even though the selection strategy for survival in high ethanol indeed generated hybrids showing superior survival in high ethanol concentrations, the method did not yield any hybrids that showed superior ethanol production, suggesting that selecting for survival in ethanol is not a suitable strategy to isolate variants that show increased fermentation performance.

We chose to solely rely on natural variation for our genome shuffling strategies and not create extra genetic variation by means of mutagenesis. This choice implies that we only work with natural alleles that have been subjected to natural selection, which might decrease the chances of introducing alleles that are beneficial for the phenotype of interest (ethanol production) but detrimental for other vital phenotypes. However, the strategy also comes with limitations. By only using natural strains, the number of beneficial mutations present in the pool might be smaller than when an extensive mutagenesis treatment is applied on the starting strains.

The results obtained with targeted crosses demonstrate that most selected outcrossed F1 hybrids showed best-parent heterosis and outperformed both of their parental strains. The superior performance of hybrids compared to their parents (best-parent heterosis) has been observed in yeast before. Systematic analyses of pairwise crosses of strains described by Liti and colleagues [31] showed that heterosis does occur, although the extent varies from study to study [32-34]. Heterosis was also shown for ethanol production [35] and ethanol tolerance [36]. Nevertheless, the exact mechanisms underlying heterosis remain largely unknown. For future work, it would be interesting to characterize the novel strains we generated with increased fermentation capacity at the genetic and phenotypic level, for example by using a QTL mapping approach as described by Duitama et al. [37].

Previous genome shuffling studies also created yeast hybrids with increased ethanol production. Many of these studies were based on the similar principles than the ones we used, that is, selecting for stress resistance after each round of shuffling and testing the obtained hybrids (often F3 hybrids) for their fermentation capacity [10,17,28,38]. Alternatively, individual fermentation trials were performed after each round of shuffling and only hybrids with increased fermentation performance advanced to the next round of genome shuffling [12,14-16,25,27]. However, most of these studies started from a mutagenized single strain, of which the initial performance is often not compared to a larger set of industrial strains. This makes it difficult to compare the results of these previous studies to those reported in this study and to estimate whether these previous studies yielded strains that could be interesting for industrial applications.

Our study yielded eight hybrids that showed a statistically higher maximal ethanol accumulation compared to Ethanol Red, which was unable to completely ferment all the available sugar in the VHG (350 g glucose per liter) medium that we used in our small-scale trials. One of these hybrids was further tested and shown to retain its superior fermentation performance on an 8-l pilot scale, making it an attractive candidate strain for commercial bioethanol production. We can imagine that our best hybrids could be further improved, using for instance directed evolution or mutagenesis and screening, to fit to the specific conditions of a particular fermentation process.

## Conclusions

In this study, we have shown that genome shuffling of natural, genetically divergent *Saccharomyces* strains can generate novel hybrids with increased ethanol tolerance and fermentation capacity. In addition, we found that selecting for growth in the presence of ethanol increases the efficiency of finding those (rare) variants that show increased fermentation performance. Although additional rounds of genome shuffling yielded some hybrids with increased fermentation capacity, on average, F1 hybrids showed higher ethanol production than F3 hybrids. The two best-performing hybrids, H1 and H2, produced approximately 1.0% (*v*/*v*) more ethanol under VHG conditions than Ethanol Red, the best-fermenting strain out of the collection and one of the parental strains. Hybrid H1 also showed an excellent fermentation capacity on a pilot scale and is therefore an interesting candidate strain for industrial applications.

## Methods

### Strains and media

In this study, we screened 318 industrial and wild *Saccharomyces* yeasts, including 26 representative (homozygous diploid) *S. cerevisiae* strains described in a previous paper [31]. The strains can be divided according to their origin as shown in Table 3.

**Table 3:** **Overview of strain collection used in this study**

**Origin**	**Number of strains**
Ale beer	132
Lager beer	46 (*S. pastorianus*)
Wine	79
Sake	14
Spirits	11
Bakery	10
Biofuels	7
Wild	19

The interdelta genetic fingerprinting assay was carried out as described in previous studies by Legras and Karst [20] and Steensels and colleagues [21].

The eight parental strains used in the different genome shuffling strategies were all diploid heterothallic *S. cerevisiae* strains and are listed in Table 4.

**Table 4:** **Overview of strains selected for genome shuffling**

**Code paper**	**Original code (origin)**
P1	Y244 (ale)
P2	Y310 (wine)
P3	Y142 (sake)
P4	Y317 (sake)
P5	Y148 (spirits)
P6	Y114 (biofuels)
P7	Y115 (Ethanol Red)
P8	Y242 (biofuels)

Yeast strains were routinely grown in YPD medium (1% (*w*/*v*) yeast extract (LabM, Heywood, UK), 2% (*w*/*v*) peptone (BD Biosciences, San Jose, CA, USA), 2% (*w*/*v*) D-glucose (Sigma-Aldrich, St. Louis, MO, USA), solidified with 2% (*w*/*v*) agar (Invitrogen, Carlsbad, CA, USA) to make plates. For long-time storage at −80°C, YPD + 25% (*v*/*v*) glycerol was used. For growth on glucose in the presence of ethanol, we used YP + 4%(*w*/*v*) glucose supplemented with ethanol to enhance the Crabtree effect. To select for antibiotic markers, solid YPD medium was supplemented with 200 μg/ml G418 (ForMedium, Hunstanton, UK), 200 μg/ml hygromycin B (Invitrogen), or both for dual selection. Sporulation was induced on minimal sporulation medium (1% (*w*/*v*) KAc, 0.05% (*w*/*v*) amino acids, 2% (*w*/*v*) agar) at 23°C after pre-growth in YPD (see also further). Tetrad dissection was carried out using a Singer micromanipulator as described [39]; mating-type determination of germinated spores was carried out by mating-type PCR [40].

### Mass sporulation, random spore isolation and mass mating

To create an F1 pool for genome shuffling with random mating, each parental strain was sporulated and subjected to random spore isolation individually (see further). Equal amounts of spores (around 6.25 × 10^6^ spores per strain, that is, 5 × 10^7^ spores in total) were mixed in 50 ml rich medium and allowed to mate randomly for 16 h. We verified that each parental strain yielded a viable spore suspension (50 to 90% viability by randomly picking 20 spores for each strain). After this period of germination, mating and proliferation (approximately six population doublings), the whole culture (F1) was split into aliquots which were frozen at −80°C. Each genome shuffling with random mating experiment was started from these F1 aliquots. To start up sporulation for populations of hybrids, cells were thawed, added to 50 ml YPD and grown for three to four population doublings after which approximately 3 × 10^8^ cells were collected and put on solid minimal sporulation medium. After 5 to 10 days of incubation, random spore isolation was carried out using a modified version of a previously described protocol [41]; all biomass from a square sporulation plate was scraped/washed off, digested overnight (35°C, 80 rpm) in 8 ml 0.1 mg/ml zymolyase (100 T, Amsbio), transferred to a falcon tube, and vortexed in the presence of approximately 400 μl sterile glass beads (150 to 212 μm, Sigma) for 3 min, and subjected to sonication (Branson Digital Sonifier, amplitude = 50%) with cooling in between as described. In the end we determined the density and purity (typically >99% pure) of spore suspensions microscopically. Mass mating was carried out in 50 ml of rich liquid medium (1% (*w*/*v*) yeast extract, 3% (*w*/*v*) peptone, 5% (*w*/*v*) glucose) by inoculating purified single spores to a starting density of 10^6^ spores/ml, followed by 16 h of incubation at 30°C, 80 rpm. After mating, cells were frozen down until further processing (ethanol tolerance assay for genome shuffling with selection or pre-growth before sporulation for genome shuffling without selection, see also Additional file 1: Supplemental Text).

### Genome shuffling with random mating and selection strategies

For random genome shuffling with growth selection, one F1 aliquot was grown overnight in 50 ml YPD, subcultured into YPD + 5% (*v*/*v*) ethanol and grown for 8 h. The OD_600_ was measured and serial dilutions were made and spread onto YPD agar plates supplemented with ethanol (10 to 12% (*v*/*v*)). The plates were sealed with parafilm and put in a plastic bag to minimize ethanol evaporation, and incubated at 30°C. The appearance of colony-forming units was monitored for up to 3 weeks. Next, we selected a plate from which yeasts were recuperated for further breeding, based on the following criteria: 1) an ethanol percentage as high as possible, 2) substantial growth on the plate (>150 CFUs), and 3) variation in colony size within the plate, indicating enrichment for the best-performing hybrids (see also Additional file 1: Supplemental Text). For replicate A, we harvested F1 hybrids from plates containing 10% (*v*/*v*) ethanol and F2 hybrids from 11% (*v*/*v*). For replicates B and C, F1 hybrids were selected from 11% (*v*/*v*) and F2 hybrids from plates containing 11.3% (*v*/*v*) ethanol. Biomass was washed off, mixed, and frozen at −80°C. For the next round of genome shuffling, an entire vial was thawed and subjected to pre-growth and mass sporulation as described. To compare ethanol tolerance between different rounds of shuffling, for each replicate and the unselected F1 pool 24, random isolates were obtained after streaking frozen populations, which were obtained after washing off the biomass after selection, on YPD agar. Each of these colonies was grown individually in liquid and had its ethanol tolerance measured using a robot-based spotting assay (see further). In this assay, values were normalized to parental strain P3, the strongest parental strain.

For random genome shuffling with selection for survival in high ethanol levels, five F1 aliquots were combined and grown in 50 ml YPD to an OD_600_ of approximately 5.0 (approximately 10^8^ cells/ml). Per survival assay, 10^9^ cells were collected, spun down, resuspended in 10 ml of selective medium (YP + 4% glucose + ethanol) and subjected to ethanol shock for 16 h (18 to 22% (*v*/*v*)). After this period, all biomass was spun down, washed with PBS, and resuspended in 1 ml PBS. Ninety percent of this biomass was plated on nine plates containing synthetic complete (SC) medium (6.7 g/l Difco™ Yeast Nitrogen Base (BD), 2 g/l SC amino acids (MP Biomedicals, Santa Ana, CA, USA)) +3% (*v*/*v*) glycerol + 2% (w/v) agar to specifically select for surviving cells that were still able to respire (non-petites). The remaining biomass was used to prepare serial dilutions which were also plated on SC + 3% (*v*/*v*) glycerol + 2% (*w*/*v*) agar. Plates were incubated for five days at 30°C after which the number of CFUs was determined. Per round of shuffling, we tested the survival capacity in a range of ethanol concentrations. By selecting biomass from a certain concentration of ethanol for the next round of shuffling, we used three selection criteria: 1) an ethanol percentage as high as possible, 2) substantial number of genetically unique hybrids (>150 unique hybrids which corresponds to >5400 CFUs; see also Additional file 1: Supplemental Text), 3) stringent selection (<1% of population should survive). We harvested F1 hybrids that were exposed to 18% (*v*/*v*) ethanol for all three replicates and F2 hybrids that survived 19% (*v*/*v*) (only for replicate C-F2 we harvested less biomass, that is, 2492 CFUs, in order to not select from the same ethanol concentration (18% (*v*/*v*)) for two subsequent rounds of shuffling). All biomass was washed off, mixed, and frozen at −80°C. For the next round of genome shuffling, an entire vial was thawed and subjected to pre-growth and mass sporulation as described.

### Genome shuffling with targeted mating and selection strategies

For targeted genome shuffling, we used a similar approach to Zheng et al. [25] to select for outcrossed hybrids. For the first round of mating, for each parental strain, we generated a pRS41H-transformed and a pRS41K-transformed strain (plasmids are described by Taxis and Knop [42]) using a standard yeast transformation protocol [43]. pRS41H and pRS41K are ARS/CEN yeast shuttle vectors harboring a resistance gene against hygromycin B (*hphNT1*) or Geneticin (*kanMX4*), respectively. We confirmed that these markers were largely retained after sporulation and germination (data not shown). For the first round of mating, the parental strains were sporulated individually, and tetrads were semi-digested using the random spore isolation protocol described above (omitting the sonication steps). These suspensions were divided over 96-well plates to generate masterplates to use for pairwise combinations of spore suspensions using the Singer ROTOR HDA^©^. Masterplates were designed to allow all pairwise matings between parental strains to take place in multiple biological replicates. Mating occurred on Singer Plus Plates filled with YPD agar, by combining approximately 10^5^ spores of each suspension on the same position of the plate using sterile RePad 96LW pins using the Singer ROTOR HDA^©^. After 24 h of mating at room temperature, we replica-plated to YPD plates supplemented with both G418 and hygromycin B, and repeated this the next day. As a negative control, we always included a mating spot in which strains with the same antibiotic resistance marker were mated. For the next round of shuffling, hybrids that were selected for ethanol tolerance (see further) or unselected hybrids were re-streaked on YPD until both markers were lost, plasmids were re-transformed, and sporulation and pairwise matings were carried out similarly as described above. Isolates that were used for a next round of shuffling were always tested for sporulation capacity and spore viability. Unselected hybrids were always taken from a random mating event without carrying out a test for ethanol tolerance.

After mating and replica-plating (see before), we subcultured F1 hybrids from solid medium into liquid YPD (96-well plate filled with 150 μl medium/well) using the ROTOR, incubated the cells overnight (horizontal shaker 900 rpm, 30°C), subcultured them the next day into fresh YPD + 5% (*v*/*v*) ethanol (selection), and froze the cells down (no selection). Then, a robot-assisted screen for ethanol tolerance was carried out (see further). After quantification, hybrid populations were selected from which single isolates were to be obtained. In order to obtain these single clones, selected populations were entirely scraped off, cultured overnight in 5 ml YPD, subcultured into 5 ml YPD + 8% (*v*/*v*) to a starting OD_600_ = 0.5 and after overnight growth serially diluted and plated for single colonies on YPD plates with 10 to 11% (*v*/*v*) ethanol. The fastest-growing colonies were re-tested for their ethanol tolerance as described before (pre-growth in YPD + 5% (*v*/*v*) ethanol followed by spotting on different ethanol concentrations). The best clone was used as a parental strain for the next round of shuffling.

### Robot-assisted screen for ethanol tolerance

To measure the ethanol tolerance of a large number of strains or hybrids in a high-throughput fashion, we used a robot-assisted spotting assay. First, strains or hybrids were freshly grown on YPD agar (approximately 50 ml Singer Plus Plate) in a 96-well format. Then, using the ROTOR, these stains were subcultured into liquid YPD (96-well plate filled with 150 μl medium/well); next, the cells were incubated overnight (horizontal shaker 900 rpm, 30°C) and subcultured the next day into fresh YPD + 5% (*v*/*v*) ethanol. Cells were grown for 2 days to saturation, after which the plate served as a source to spot cells on multiple solid Singer Plus Plates containing YPD supplemented with various levels (10 to 13% (*v*/*v*)) of ethanol using the ROTOR equipped with RePad 96LW pins. After spotting, plates were incubated at 30°C (sealed with parafilm and put in a plastic bag) and scanned using an Epson Perfection V700 PHOTO at 300 dpi at regular intervals, typically for up to 2 to 3 weeks. YPD plates (control) were scanned after two days. The scans were quantified in ImageJ using the ScreenMill software suite operated in summation mode [44]. For screening natural strains, ethanol tolerance was expressed as the end point area on medium with ethanol normalized by the area on YPD. For screening hybrids during genome shuffling with targeted mating, this YPD-normalized area was normalized by the area of a strong parental strain on the same plate (P2).

### Lab-scale VHG fermentations

Lab-scale fermentations under VHG conditions were started with an overnight pre-growth of a single colony into 3 ml YPD, followed by a transfer of the entire culture to 30 ml YP + 4% (*w*/*v*) glucose and additional growth for 48 h to the stationary phase (200 rpm, 30°C); 250-ml Schott bottles each filled with 150 ml YP + 35% (*w*/*v*) glucose and a magnetic rod (35 × 5 mm) were inoculated to a starting OD_600_ = 1.0 (approximately 2.0 × 10^7^ cells/ml). These bottles were sealed with a waterlock and stirred continuously at 150 rpm on a magnetic stirring platform (IKA® RO 15) at 30°C. The bottles were weighed on a daily base to determine the cumulative weight loss, a proxy for CO_2_ production. Each fermentation was terminated when its weight loss dropped below 0.20 g/day. In each fermentation batch, we included three biological replicates of Ethanol Red as a control strain; the ethanol production of each tested strain was subsequently expressed relative to the average ethanol production of these three control fermentations. The screening of the yeast collection for VHG fermentation capacity was carried out similarly; however, fermentations were kept static and were terminated after 14 days. We found a good correlation (Spearman *r* = 0.85, *P* ≤ 0.001) between the ethanol level reached in static and stirred fermentations (Additional file 1: Table S1).

Fermentations of pools of hybrids were carried out under stirred conditions as described above, but started by thawing a vial of hybrids and adding it completely to 30 ml YP + 4% (*w*/*v*) glucose for pre-growth. Re-inoculation took place by harvesting two thirds of all the biomass and inoculating it directly into fresh VHG medium. The ethanol production was determined using Anton Paar Alcolyzer and the end glucose concentration using a commercial GOD-PAP assay (Dialab).

### VHG fermentations under semi-industrial conditions

To pre-screen the strains under semi-industrial VHG conditions, each strain was grown overnight in 5 ml YPD, subcultured by transferring 1 ml of this culture to 10 ml YP + 5% (*w*/*v*) glucose and grown for 3 days, after which 10 ml of this culture was added to 100 ml YP + 10% (*w*/*v*) glucose, which was grown overnight. Then, after washing one time with Ringer solution, a biomass suspension containing 50 g wet biomass in 100 ml Ringer solution was created. Next, bottles containing 100 ml VHG medium (2% (*w*/*v*) peptone, 2% (*w*/*v*) malt extract, 0.12% (*w*/*v*) Fermaid, 109.9 mg/ml ZnSO_4_.7H_2_O, 320 g/l glucose, antifoam) were inoculated with 1 ml of this biomass suspension (approximately 0.5 g wet biomass). The fermentations were kept static at 30°C and terminated after 7 days after which the ethanol concentration was determined using Anton Paar Alcolyzer and HPLC.

To determine the performance of the best strains of the pre-screening on a pilot scale (8 l), strains were grown overnight in 5 ml YPD, subcultured by transferring the entire culture to 150 ml YP + 5% (*w*/*v*) glucose followed by 1 day of growth. Then, this entire culture was added to 2 l YP + 10% (*w*/*v*) glucose which was grown for three days. Biomass was washed one time with Ringer solution, after which a biomass suspension containing 50 g wet biomass in 100 ml Ringer solution was created. Sartorius Biostat B plus fermentors filled with 8 l VHG medium were inoculated with 80 ml biomass suspension. This medium was aerated for 30 min pre-fermentation; the pH was kept at 4.5 as soon as it reached this value by addition of 12% ammonia. Fermentations were stirred at 300 rpm for the first 4 h after which the stirring speed was adjusted to 50 rpm for the remaining time. Fermentations were terminated after 7 days.

### Data analysis

Genetic fingerprints were analyzed using BioNumerics (Applied Maths, Belgium) and clustered according to the dice correlation coefficient to build a similarity matrix and a Unweighted Pair Group Method with Arithmetic Mean (UPGMA) algorithm. Heat maps were created by converting VHG ethanol production data to *Z*-scores; sporulation, spore viability, and ethanol tolerance data were normalized to values between 0 and 1.

Statistical analyses were carried out using Graphpad Prism.

## Additional files

Additional file 1:
**Supplemental Figures, Tables and Text.**
Additional file 2:
**Fermentation data hybrids generated with genome shuffling with random mating.**
Additional file 3:
**Fermentation data hybrids generated with genome shuffling with targeted mating.**
Additional file 4:
**Fermentation data hybrids that were tested in biological triplicates.** Promising hybrids (see Additional files 2 and 3) were subjected to VHG fermentations. For each parameter the mean and standard deviation are shown. Yield was calculated based on the maximal amount of ethanol that could be formed based on glucose consumption. It is also indicated whether the ethanol production is significantly different from the ethanol production by Ethanol Red in the same batch (t-test). Additional file 5:
**Performance of hybrid H1 and 26 additional strains in small-scale VHG fermentations under static conditions.** Indicated is the average ethanol production with standard deviation and the number of biological replicates this is based on. It is also indicated whether the ethanol production significantly differs from the ethanol production of hybrid H1 (t-test).

## Abbreviations

EtOH: ethanol
OD: optical density
SC: Synthetic complete
VHG: Very high gravity
YPD: Yeast extract peptone dextrose
QTL: Quantitative trait locus
