# Supplementary material for: Large-scale robot-assisted genome shuffling yields industrial Saccharomyces cerevisiae yeasts with increased ethanol tolerance
Source: Biotechnol Biofuels. 2015 Feb 26;8:32. doi: 10.1186/s13068-015-0216-0 (PMC4354739; doi:10.1186/s13068-015-0216-0)
Supplement: Additional file 1: — Supplemental Figures, Tables and Text. [file 13068_2015_216_MOESM1_ESM.pdf]

Additional File 1: Supplemental Figures, Tables and Text

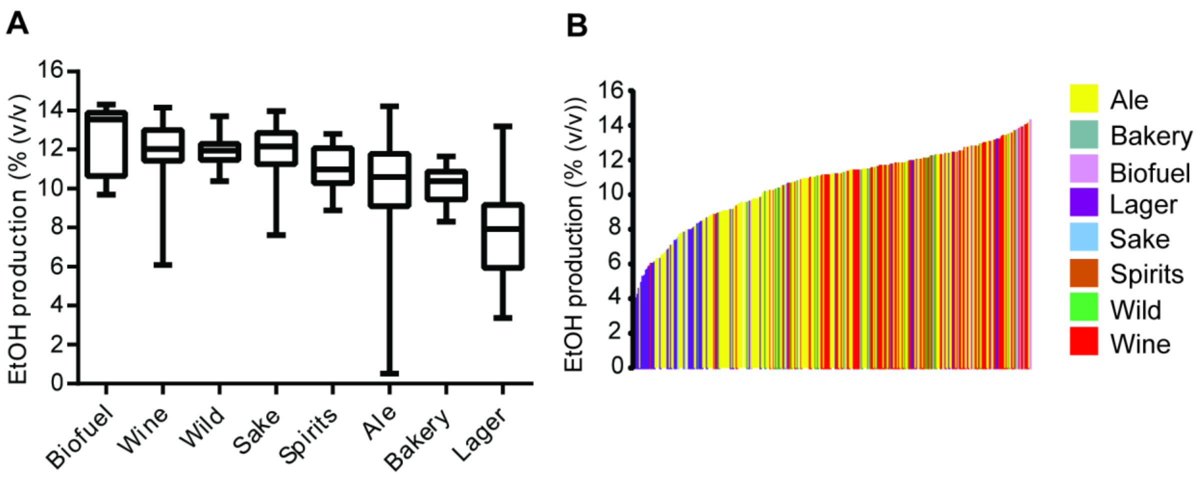

Fig. S1: *Saccharomyces* yeasts vary widely in maximal ethanol accumulation in small-scale VHG fermentations. Each strain was inoculated into 150 ml YP+35% (w/v) glucose and fermentations were kept static for 14 days, after which the final ethanol concentration was measured. (A) Boxplots showing the ethanol production of strains from different origins. (B) Strains were ranked by their ethanol accumulation and colored according to their origin.

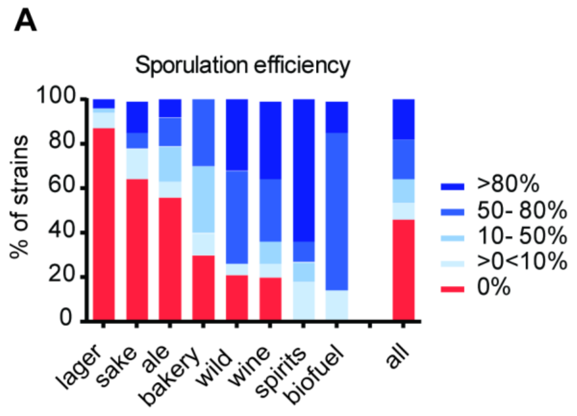

Number of strains per category:

lager	46
sake	14
ale	132
bakery	10
wild	19
wine	79
spirits	11
biofuel	7
all	318

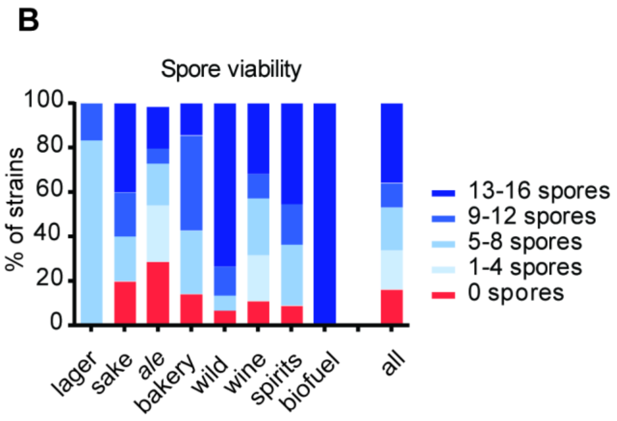

Number of dissected strains per category:

lager	6
sake	5
ale	58
bakery	7
wild	15
wine	63
spirits	11
biofuel	7
all	172

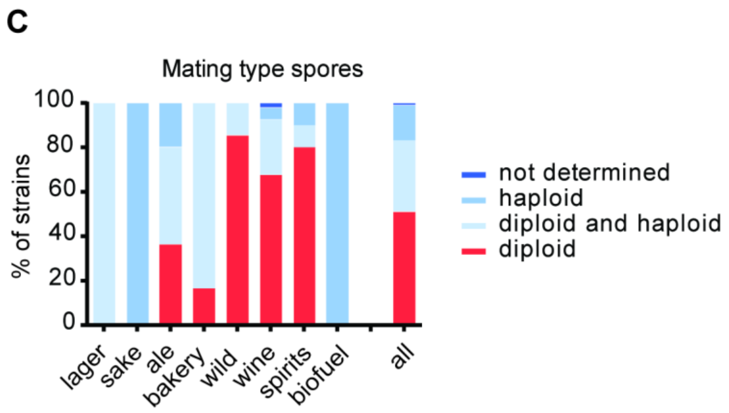

Number of strains yielding viable spores per category:

lager	6
sake	4
ale	41
bakery	6
wild	14
wine	56
spirits	10
biofuel	7
all	144

Fig. S2: *Saccharomyces* yeasts vary in sporulation efficiency and spore viability. (A) The sporulation of each strain was induced on minimal sporulation medium and the sporulation efficiency, expressed as the percentage of tetrads, was determined by light microscopy. (B) After dissecting four full tetrads, the spore viability was expressed as the fraction of spores capable of forming a colony on solid medium. Strains were divided into five bins based on their spore viability. (C) The mating-type of germinated spores was determined, and each strain was classified as yielding haploid spores, diploid and haploid spores or diploid spores.

Table S1: The ethanol production of industrial strains in static and stirred VHG fermentations. Thirteen strains, the eight parental strains as well as five additional high-ethanol producing strains, were tested for their fermentation capacity under both static and stirred conditions in 150 ml YP+35% (w/v) glucose. Under stirred conditions, each strain was tested in biological triplicates, except for P7 (Ethanol Red) which was tested in each fermentation batch (10 batches in total), and strain P4 which was tested twice in triplicates. Static fermentations were carried out once.

		**ethanol stirred (% (v/v))**		**ethanol static (% (v/v))**
**strain**	**industry**	average	stdev	
P7	biofuel	19.0	0.40	14.3
P4	sake	18.4	0.42	14.0
Y138	sake	18.5	0.37	13.8
Y116	biofuel	18.1	0.07	13.9
Y111	biofuel	18.0	0.14	13.8
P8	biofuel	17.5	0.27	12.7
P1	ale	17.1	0.02	12.4
Y113	biofuel	16.9	0.11	9.7
P6	biofuel	16.9	0.23	10.6
Y112	biofuel	16.5	0.05	13.5
P2	wine	16.4	0.16	12.3
P3	sake	15.9	0.06	12.0
P5	spirits	14.8	0.22	8.9

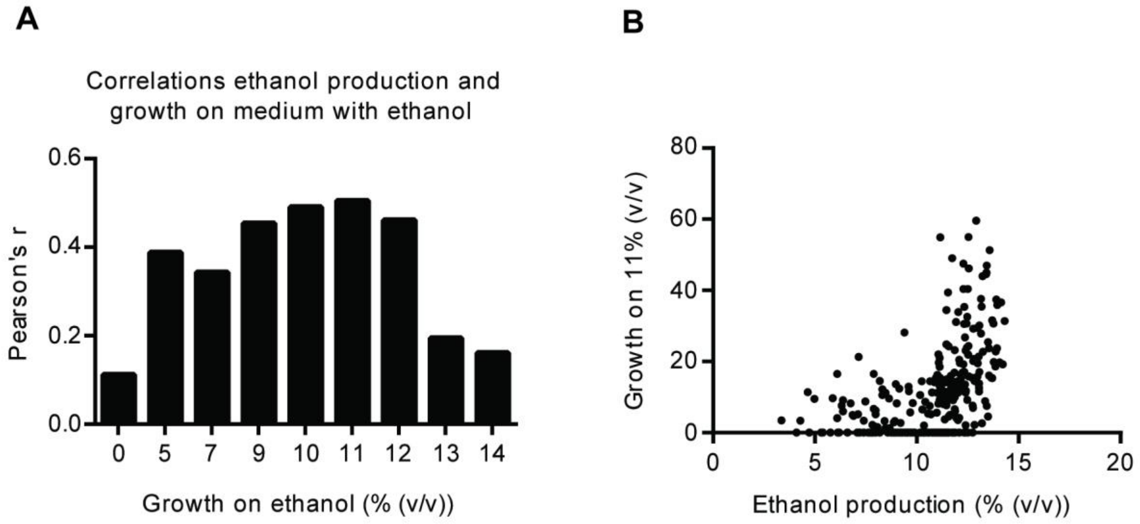

Fig. S3: The growth capacity on solid rich medium supplemented with ethanol is weakly correlated with ethanol production in a lab-scale VHG fermentation. For 308 strains used in this study both the ethanol production under static conditions and the growth area on solid medium with different concentrations of ethanol was determined (see Methods). (A) The Pearson correlation coefficient for each growth data set correlated to ethanol production indicates that these phenotypes show a weak positive correlation, which is the highest for growth on 11% (v/v) ethanol. All correlations were significant (p<0.05). (B) Example of a scatterplot of 11% (v/v) ethanol growth data against ethanol production data.

Table S2: Data genome shuffling with growth selection. Per generation and per replicate the ethanol concentration from which biomass was collected and the number of CFUs is indicated. Also the average ethanol tolerance of 24 random clones obtained after re-testing is indicated for each replicate (endpoint area of colony on ethanol normalized by area on YPD and strongest parental strain, see Methods).

Replicate	Generation	Selection		Re-screening average norm. growth capacity (n=24)		
		**CFUs**	**ethanol (% (v/v))**	**10%**	**11%**	**12%**
	F1	NA	NA	0.40	0.26	0.09
A	F1	1 768	10	1.16	1.05	0.69
B	F1	1 168	11	1.10	0.94	0.83
C	F1	1 360	11	1.06	0.97	0.87
A	F2	1 656	11	0.85	0.77	0.55
B	F2	628	11.3	1.13	1.07	0.91
C	F2	252	11.3	0.92	0.87	0.74

Table S3: Data genome shuffling with selection for survival in high ethanol levels. The number of survivors (CFUs) per generation after exposure to rich medium supplemented with high ethanol levels able to respire is displayed (see Methods). For the first round of shuffling, cells were selected that survived 18% (v/v) ethanol exposure, for the second round of shuffling, selected cells survived 19% (v/v) ethanol (selected populations indicated in bold).

Replicate	Generation	Ethanol (% (v/v))				
		**18**	**19**	**20**	**21**	**22**
A	F1	**30 000**				
B	F1	**23 270**	1311	1		
C	F1	**16 922**	542	1		
A	F2	38 613	**5 907**	290		
B	F2	350 000	**192 000**	2 400		
C	F2	46 560	**2 492**	72		
A	F3	2 750 000	1 320 000	136 000	7 900	352
B	F3	1 720 000	406 000	21 400	1 111	5
C	F3	5 760 000	2 310 000	205 000	4 805	87

Table S4: Growth- and unselected F3 populations show poor survival capacity. The number of survivors (CFUs) per generation after exposure to rich medium supplemented with high ethanol levels is displayed.

Selection strategy	Replicate	Ethanol (% (v/v)) 18	19	20	21	22
Growth selection	A	1575	25	5	0	0
	B	14150	830	1500	0	0
	C	2295	320	36	0	0
No selection	A	530	46	0	0	0
	B	200	4	0	0	0
	C	4996	213	5	0	0
	D	2533	167	12	0	0
	E	6987	335	20	0	0
	F	4013	296	51	0	0

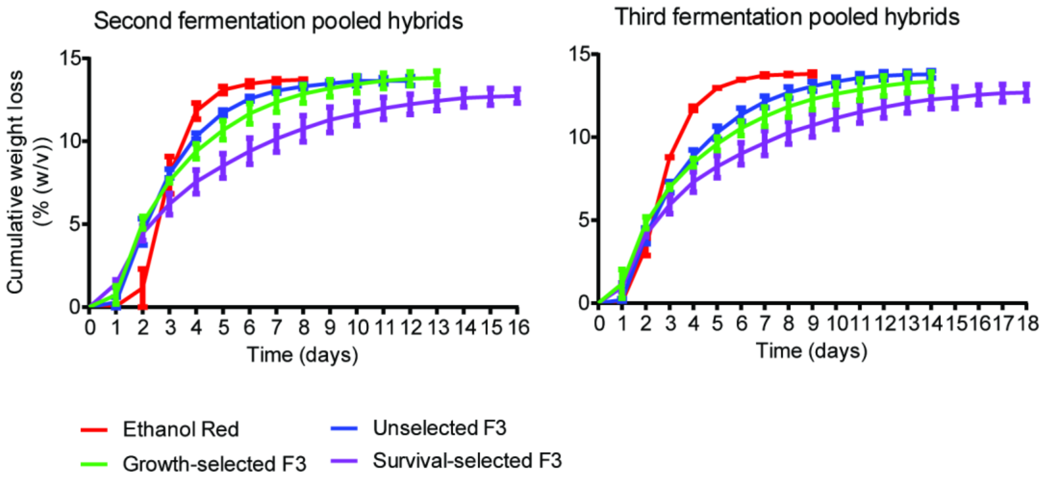

Fig. S4: Fermentation performance of F3 populations in VHG medium after re-inoculation. F3 populations obtained after random genome shuffling with different selection methods (growth, survival, no selection) were inoculated into YP+35% (w/v) glucose and re-inoculated two times (left; first re-inoculation, right; second re-inoculation). The cumulative weight loss is a measure of CO_2 production during the fermentation. Each line represents the average of two replicates (Ethanol Red), six replicates for growth-selected and survival-selected populations or twelve replicates for unselected populations. Error bars represent standard deviations.

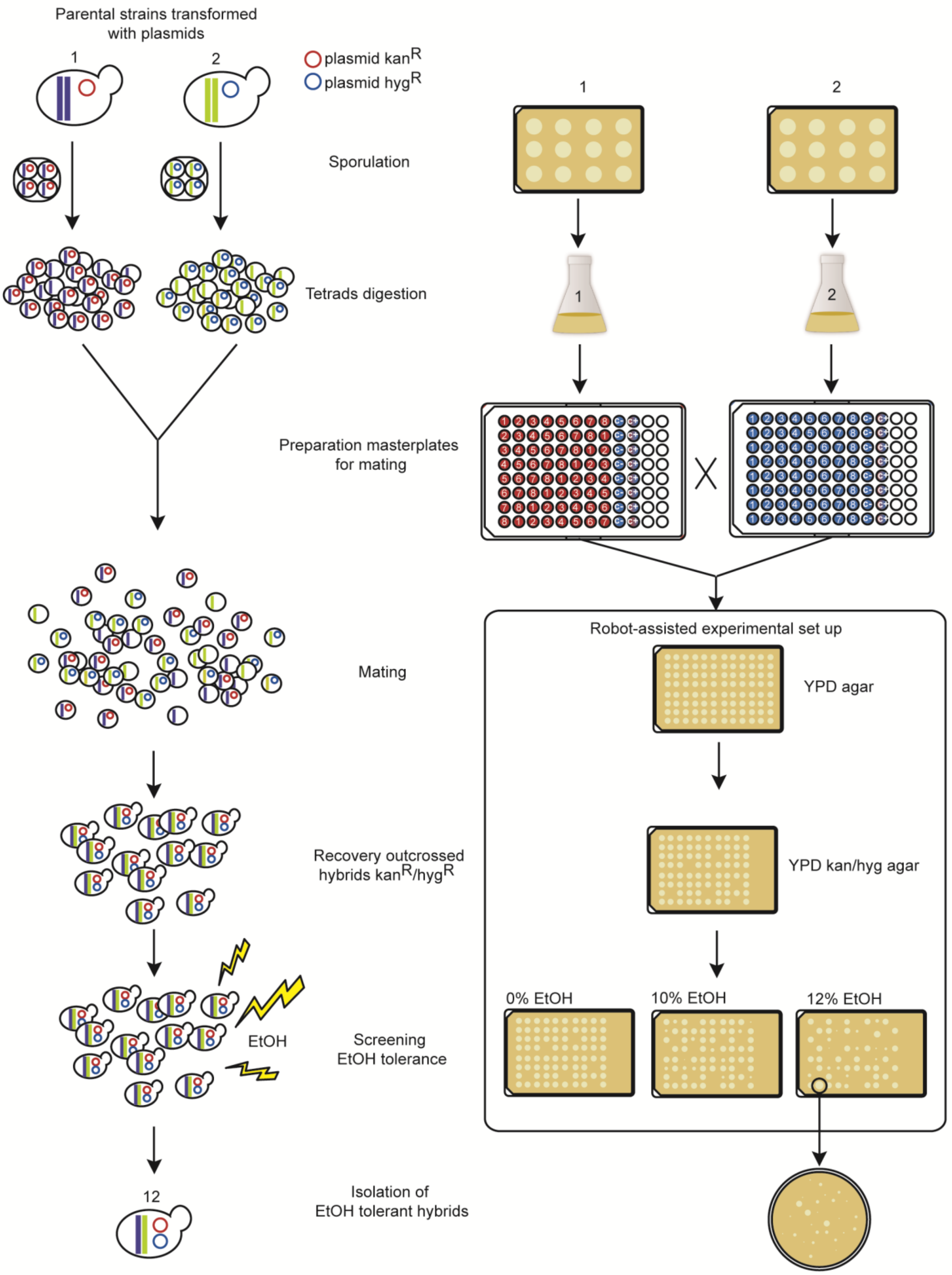

Fig. S5: Technical details of genome shuffling based on targeted mating. The left panel shows schematically the targeted mating strategy, whereas the right panel shows the technical procedure. As an example, in this figure we illustrate the targeted mating of strains P1 and P2 and the subsequent selection of an ethanol-tolerant hybrid of these strains. Each parental strain was transformed with a plasmid harboring resistance against kanamycin (kan) or hygromycin (hyg), followed by sporulation on solid sporulation medium, collection of the biomass and overnight tetrad digestion (see Methods).

Then, the digested tetrad suspensions were divided over 96-well plats to generate masterplates that were subsequently used in a robot-assisted set-up to allow for mating. In this figure two examples of these masterplates are shown. The parental strains were crossed in all pairwise combinations by placing digested tetrad suspensions in close proximity on YPD agar using the robot. After mating, outcrossed hybrids, possessing resistance against both antibiotics, were recovered by replica-plating on YPD+hyg/kan. These hybrid pools were subcultured in liquid medium and subjected to a robot-assisted ethanol tolerance screen on solid YPD supplemented with different ethanol levels as described in the Methods. After quantification, the best-performing populations were plated for single colonies on YPD supplemented with ethanol, and the fastest-growing colonies were re-tested for ethanol tolerance; the best-performing isolates were then used for the next round of shuffling, according to the same procedures (transformation of plasmids, sporulation, crossing). The crossing schemes are outlined in Figure 4. Hybrids for unselected genome shuffling schemes were isolated directly after the mating step, without carrying out any test for ethanol tolerance (not shown).

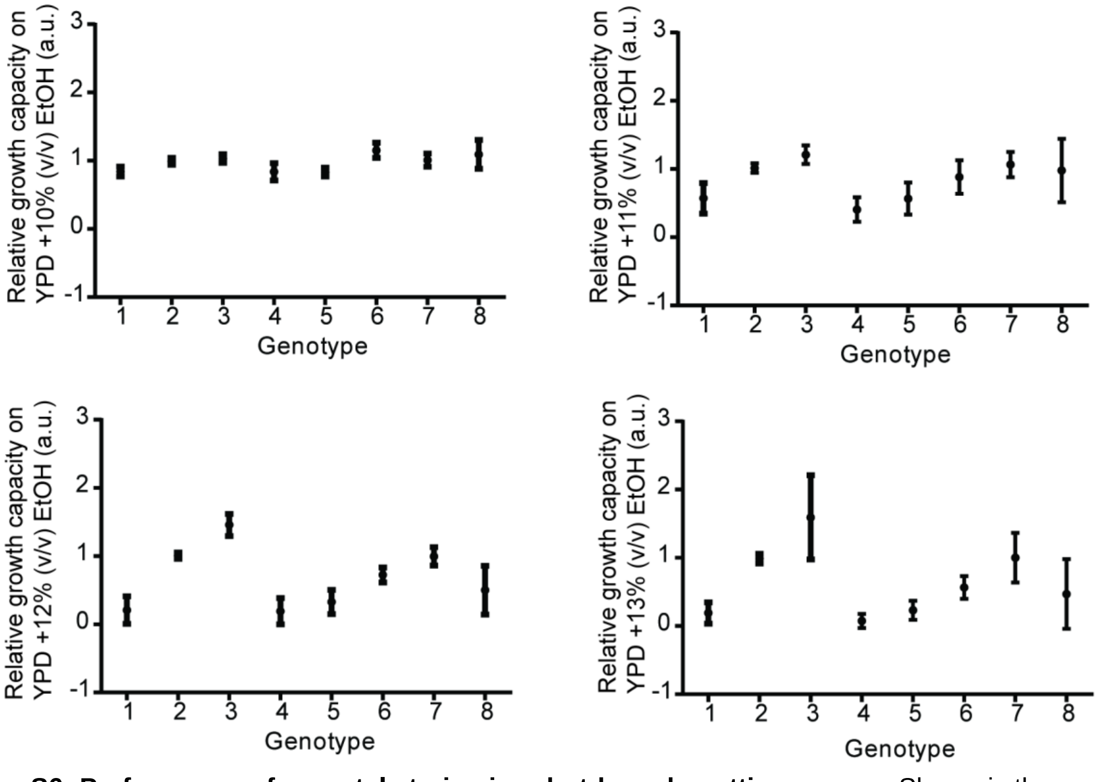

Fig. S6: Performance of parental strains in robot-based spotting assays. Shown is the average and standard deviation of the normalized colony area of each parental strain (32 data points) on YPD supplemented with different ethanol concentrations (see Methods). Parental strains are numbered 1-8.

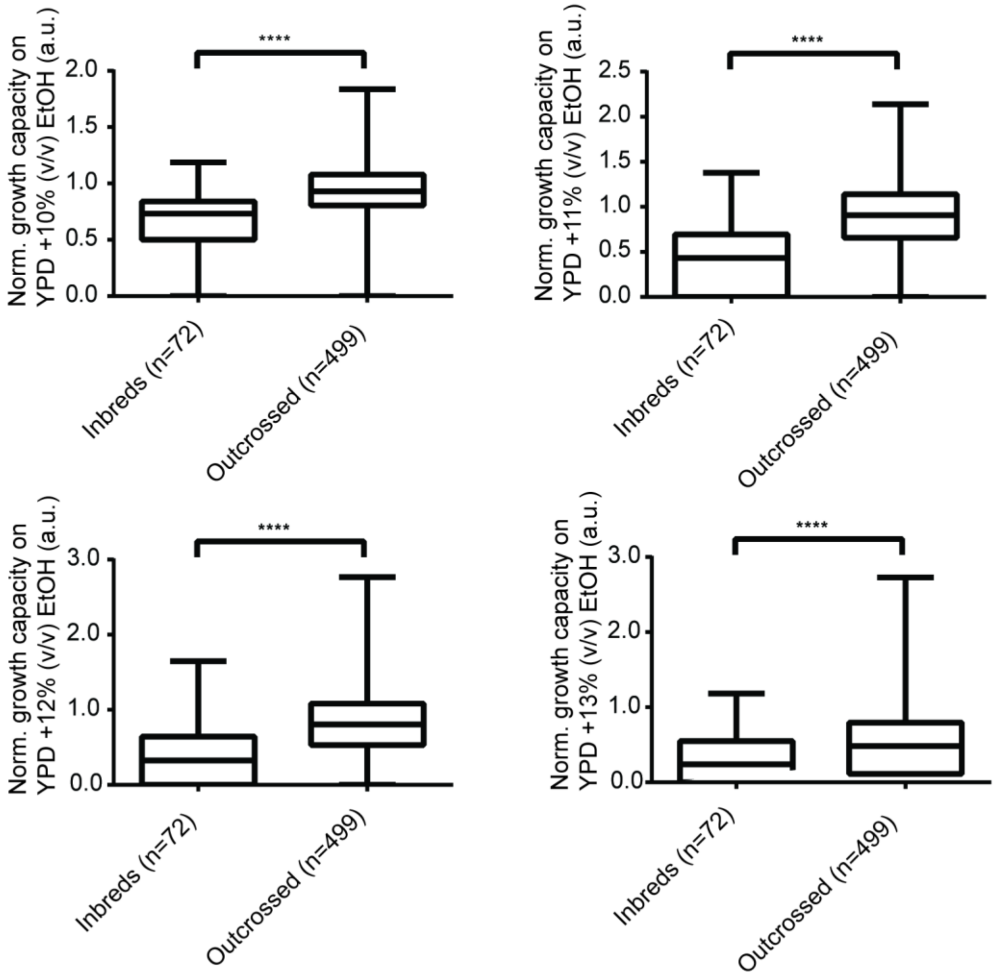

Fig. S7: Outcrossed F1 hybrid populations show statistically higher ethanol tolerance (Mann-Whitney test *p*<0.0001) on all tested concentrations than inbred F1 populations.

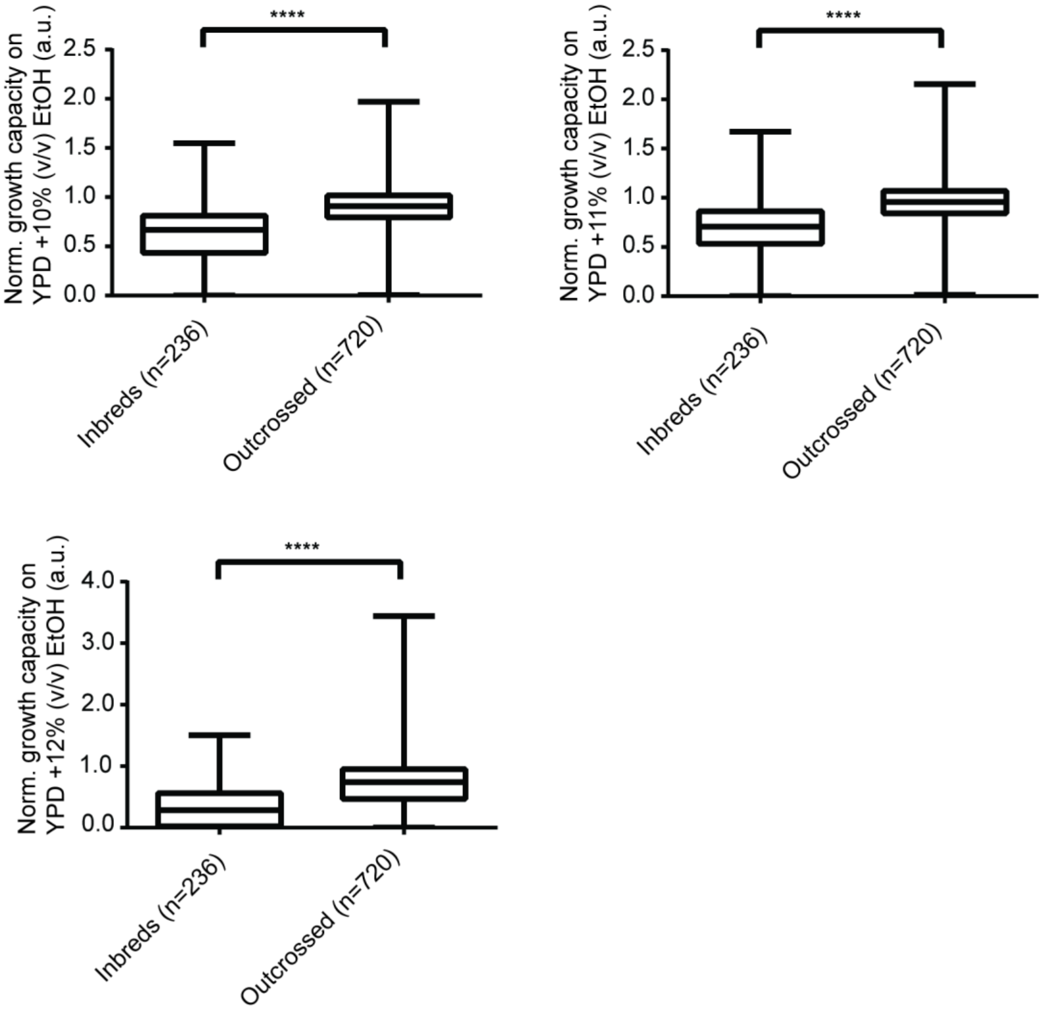

Fig. S8: Outcrossed F2 hybrid populations display higher ethanol tolerance than F2 inbred populations (Mann-Whitney test *p*<0.0001).

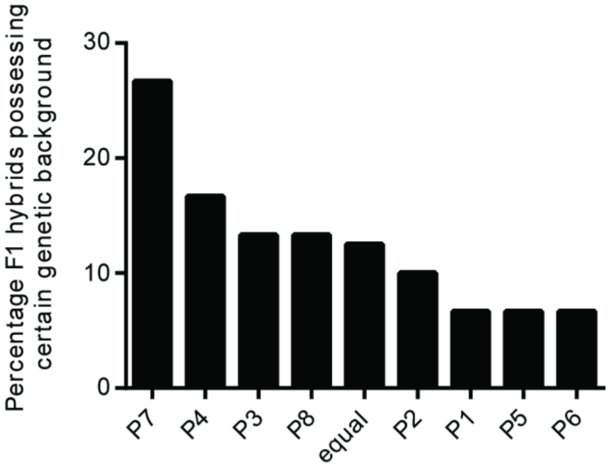

Fig. S9: Targeted F1 hybrids are enriched for P7 (=Ethanol Red) background. Overview of the contribution of each parental genome to targeted F1 hybrids selected for VHG fermentations. The percentage of the 15 F1 hybrids possessing a certain background is indicated, the "equal" bar indicates the percentage that each strain would contribute in case parental strains would contribute equally to these F1 hybrids.

Supplemental Text

This supplemental text describes details of the different genome shuffling strategies. More information can be found in the main text.

Genome shuffling with random mating to generate pool of F1 hybrids

The mass mating procedure was started with $6.25x10^6$ spores of each strain (i.e. a total of $5x10^7$ spores). These spores were mixed in rich medium and allowed to germinate, mate and proliferate for 16h. After 16h, the cell density had increased to ~$2x10^9$ cells. Since we used diploid heterozygous parental strains, we assume each spore is genetically unique and, because the heterothallic nature of the strains avoided haplo-selfing of germinated spores, each hybrid formed is likely unique as well. Assuming that all the spores first mated to form $2.5x10^7$ diploids, and subsequently all the resulting diploids started proliferating, after the 16h incubation the population had undergone ~6.3 doublings; i.e. each unique diploid was present in ~80 copies at the end. The whole population was divided into aliquots which were frozen down at -80 °C; each aliquot harboring ~$1.1*10^7$ cells (0.58% of the entire population). Hence, we can expect that each F1 aliquot contains 0.0058x80=0.47 copies of each unique hybrid.

Random genome shuffling with growth selection

In order to select cells for the next round of shuffling during random mating strategy with selection for growth in the presence of ethanol, we plated dilution series on YPD plates supplemented with a range of ethanol concentrations. For the first round of selection, for each biological replicate, we started from a single F1 aliquot. We grew these cells in 50 ml YPD overnight, typically for n~7. After this overnight growth, typically 1.5-10% of the culture was subcultured in YPD+5%(v/v) ethanol and allowed to grow for 8h, and if we assume each initial hybrid was unique and grew with the same growth rate, we would obtain ~135 copies of each unique hybrid before plating. By plating serial dilutions, only a fraction of unique hybrids were present on a given plate. We always harvested a 10^{-2} or 10^{-3} dilution for the next round of shuffling; which implies that we subjected ~0.2 – 0.02% of all unique hybrids to selection. After incubating the plates, cell growth was inspected, and we always took at least 150 unique hybrids (see Methods) to the next round of shuffling. After washing off and mixing the biomass, cells were frozen down, and the start for sporulation was carried out as described in Methods. After sporulation and mass mating similar population size was frozen down and the same assumptions were made as before.

Random genome shuffling with survival selection

In order to select cells for the next round of shuffling during random mating strategies with selection for survival of very high ethanol levels, we incubated cell populations for 16h in YPD supplemented with high ethanol concentrations. For the first round of selection, for each biological replicate, we started from five F1 aliquots. We pre-grew these cells in 50 ml YPD to an OD600~5 (~$1.0*10^8$ cells/ml), corresponding to n~7. If we assume at the start every unique hybrid was present twice, the number of each unique hybrid (assuming equal growth rates in YPD) would have increased to ~181. Then, per assay, $1.0*10^9$ cells (~20% of the pre-culture) were harvested, i.e. ~36 copies of each unique hybrid were tested in each assay. Although there was 4% (w/v) glucose in this medium, the OD_{600} after 16h did not change significantly, indicating that at these high ethanol levels cell proliferation was inhibited. As a control, a portion of the culture was incubated in YPD without added ethanol, always undergoing 2-3 population doublings. After 16h incubation, we spun down the culture immediately, washed 1x with PBS, and resuspended the cells in a total volume of 1 ml PBS. 90% of this biomass was spread over nine petri plates containing SC+3%(v/v)+2%(w/v) agar, the remaining biomass was used for serial dilutions that were also plated. After incubating the plates, we always took at least 150 unique hybrids to the next round of shuffling (see Methods). After washing off and mixing the biomass, aliquots were created and frozen down, and the start for sporulation was carried out as described in Methods. After sporulation and mass mating the same assumptions were made as described above.
